# A phase I study of pazopanib in combination with escalating doses of ^131^I in patients with well-differentiated thyroid carcinoma borderline refractory to radioiodine

**DOI:** 10.1371/journal.pone.0178325

**Published:** 2017-06-29

**Authors:** Laura Q. Chow, Rafael Santana-Davila, Austin Pantel, Mara Roth, Leslie N. Anderson, Alan Failor, Robert Doot, David Mankoff

**Affiliations:** 1 Department of Medicine, Division of Medical Oncology, University of Washington, Fred Hutchison Cancer Research Center, Seattle, WA, United States of America; 2 Department of Nuclear Medicine, University of Pennsylvania, Philadelphia, PA, United States of America; 3 Department of Medicine, Division of Endocrinology, University of Washington, Seattle, WA, United States of America; Cardiff University, UNITED KINGDOM

## Abstract

**Objective:**

This trial was conducted to evaluate the ability of pazopanib to overcome therapeutic ^131^I resistance.

**Materials, methods and patients:**

This phase 1 trial assesses the combination of pazopanib and escalating doses of radioiodine (^131^I) in patients with recurrent or metastatic thyroid cancer that are borderline or relatively iodine refractory. Radioiodine uptake scans were assessed post therapy and compared to historical pre-treatment scans. Patients underwent FDG PET/CT before and after the initial pazopanib treatment to identify the impact of pazopanib on the cancer prior to ^131^I therapy.

**Results:**

A dose limiting toxicity (cardiac arrhythmia and grade 3 fatigue) in the first patient in the first cohort prompted expansion to a total of 6 patients. Additional grade 3–4 hematologic toxicity and low accrual in the expanded cohort led to the decision not to pursue further study of the regimen. In patients with measurable disease 4/5 (80%) achieved stable disease. Median progression free survival was 6.7 months. At 3 years of follow up, one patient died due to progressive disease, two are being treated with systemic therapy and 3 continue without requiring subsequent therapy at 15, 27 and 35 months from the last dose of pazopanib. There was no convincing impact of pazopanib on iodine uptake in scans performed pre- and post-therapy compared to scans from historical ^131^I treatments without pazopanib.

**Conclusion:**

Despite a suggestion of therapeutic efficacy, combined pazopanib and ^131^I resulted in increased toxicity. There was no convincing evidence that the administration of pazopanib improved iodine uptake or retention.

**Trial registration:**

ClinicalTrials.gov NCT01413113

## Introduction

As the most common endocrine malignancy, thyroid cancer comprises 1–2% of all cancers in the United States, and its incidence has doubled in the last decade.[1,2] In 2015, an estimated 62,450 patients will be diagnosed with thyroid cancer in the United States, and the disease will be responsible for 1,950 deaths.[3] Most patients with localized well-differentiated thyroid carcinoma (WDTC) are successfully cured by surgery followed by radiation therapy. However, 10–15% of patients with WDTC develop recurrent disease, distant metastatic disease, or both.[4] For patients with recurrent or metastatic WDTC who have a papillary or follicular subtypes, radioactive iodine (^131^I) (RAI) has been the treatment of choice since the 1940s, as the neoplastic cells retain the thyroid’s unique ability to absorb iodine. ^131^I therapy allows the targeted delivery of relatively high doses of radiation to the tumor with only modest systemic toxicity which makes it the most successful therapy for this condition.[5] The use of radioiodine dosimetry methods to calculate the maximally tolerated dose that can be administered in individual treatments has been shown to improve response at acceptable toxicity levels, especially for metastatic disease.[6,7]

Most RAI-avid WDTC patients respond well to ^131^I therapy, except patients with bulkier tumors and those with predominant bone disease, Ultimtely, however, most patients with persistent disease develop iodine resistance while others may reach cumulative levels of ^131^I exposure that can lead to unacceptable bone marrow toxicity and an elevated risk of leukemia. The options of treatment after ^131^I are limited, and survival is greatly decreased. Chemotherapy has been ineffective against WDTC and the standard of care developed over the last decades is the use of several multi-targeted anti-angiogenic tyrosine kinase inhibitors (TKIs) that have been found to be efficacious [8–12] The mechanisms of action of these TKIs against thyroid cancer are incompletely understood; however, all TKIs currently available inhibit multiple kinases and share a number of common targets which include the angiogenic receptors VEGFRs, PDGFRs, FGFRs, as well as RET. These agents are not well tolerated and have substantial toxicities that limit their long term administration and substantially affect the patient’s quality of life.

Some components of iodine resistance that have been identified include aberrant activation of the MAPK and/or the PI3K-AKT pathways that leads to the impairment of the iodide-handling machinery and translates into ineffective radioiodine treatment[13] The combined actions of the multi-targeted anti-angiogenic TKI and concurrent ^131^I radioiodine may additively or synergistically overcome therapeutic ^131^I resistance in recurrent or metastatic well-differentiated thyroid carcinoma (WDTC) patients by (1) improving delivery of ^131^I in bulky tumors by vascular normalization, by (2) increasing sensitivity to the radiation effects, and/or by (3) independent, combined anti-tumor effects.

Pazopanib (GW786034, GlaxoSmithKline, Philadelphia, PA, USA) is an oral, multitargeted, tyrosine kinase inhibitor that has been studied in the treatment of WDTC. In a phase 2 study, 18 of 37 (49%) patients with WDTC demonstrated confirmed partial responses to pazopanib with a median PFS of 11·7 months. [9] Through its inhibition of VEGF, pazopanib inhibits tumor angiogenesis. We hypothesized that anti-angiogenic agents such as pazopanib might be synergistic with ^131^I in WDTC as (1) they have independent anti-tumor effects, (2) the anti-angiogenic effect of pazopanib might transiently normalize tumor vasculature, leading to improved radioiodine delivery and/or reduction of intratumoral hypoxia which could enhance ^131^I efficacy, [14,15]and 3) the impact of pazopanib on other target gene products might work synergistically to enhance apoptosis induced by ^131^I radiation.[16]

Guided by the hypothesis that the combined actions of the multi-targeted tyrosine kinase inhibitor (TKI), pazopanib, and concurrent ^131^I radioiodine would possibly additively or synergistically overcome therapeutic ^131^I resistance in recurrent or metastatic WDTC, we conducted a phase I trial of pazopanib at standard fixed doses in combination with escalating doses of ^131^I in patients with WDTC. Because of concern about the possible added toxicities of pazopanib and ^131^I, especially on bone marrow suppression, we conducted this phase I trial to primarily assess safety and to secondarily explore the effects on iodine update and efficacy.

## Methods

### Patient population

Adult men and women were considered for study entry if they had unresectable recurrent and/or metastatic, histologically confirmed WDTC. Patients included in the study were required to have at least some evidence of tumor iodine uptake on either diagnostic or post-therapy radioiodine scans and ^131^I refractory/resistant as defined as one of the following within 12 months of ^131^I therapy: 1) one or more measurable lesions and evidence of disease progression by RECIST 1.1 or 2) persistent disease and >50% increase in suppressed thyroglobulin levels. In addition, patients were required to have some evidence of disease that might benefit from enhanced iodine uptake and efficacy that included 1) low or absent pre-therapy iodine uptake in at least one measureable lesion or 2) evidence of disease progression (as defined above) despite evidence of iodine uptake on pre- or post-therapy iodine scanning. Additional entry criteria included an East Cooperative Oncology Group (ECOG) performance status of 2 or better and adequate organ function (i.e., hematologic, hepatic, renal). Patients with and without liver metastases could be included. For patients without liver metastasis, levels of alanine aminotransferase (ALT) and aspartate aminotransferase (AST) needed to equal ≤ 2.5 times the upper limit of normal (ULN). For patients with liver metastasis, ALT and AST needed to equal < 5 times the ULN. Patients were excluded if there were previously treated with cumulative ^131^I exposure in excess of 1000 mCi, Additional exclusion criteria were patients with the following conditions: poorly controlled hypertension; clinically significant cardiovascular disease; symptomatic involvement of the central nervous system; active gastrointestinal fistula, ulceration, perforation, abscess, clinical bleeding, and/or gastrointestinal malabsorption; pregnancy or lactation; and unwillingness or inability to give informed consent. As is typical in this patient population, no iodine contrast administration was allowed within a month before the administration of ^131^I therapy.

The trial was conducted at the Seattle Cancer Care Alliance and was approved by the IRB at the University of Washington and conducted in accordance with the International Conference on Harmonization Good Clinical Practice guidelines, and applicable local laws and regulatory requirements. Patients were required to understand and sign an informed consent before pariticipating in the study.

### Study design

This was a single-center phase I, open-label, dose-finding, safety study of pazopanib in combination with ^131^I in patients with advanced or recurrent WDTC refractory to ^131^I as defined above. The trial used a fixed dose of pazopanib previously shown to be safe and effective in prior phase 2 trials[8] and an escalating dose of ^131^I defined by blood and marrow exposure levels estimated by standard methods for radioiodine dosimetry.[6,7]

Dose cohorts followed a standard phase I 3+3 design[17] (Table 1), starting at a 25 rad estimated blood/marrow dose and designed to escalate to 150 rads, which was slightly below the maximum dose recommended maximum blood/marrow dose for iodine alone– 200 rads.[7] No intrapatient dose escalation was allowed.

**Table 1:** Dose levels for pazopanib in combination with ^131^I.

Dose Level	Pazopanib dose, mg^a^	^131^I dose, rad
-2	600	25
-1	600	50
1 (starting dose)	800	50
2	800	100
3	800	125
4	800	150

^a^ administered orally, once daily

The operating characteristics of this study design are shown in Table 2, which provides the probability of escalation to the next higher dose for each underlying true DLT rate. For example, for a toxicity that occurs in 5% of subjects, there is a greater than 95% probability of escalating. Conversely, for a common toxicity that occurs with a rate of 70%, the probability of escalating is <5%. Table 2 also shows the probability of failing to observe toxicity in a sample size of 3 or 6 patients given various true underlying toxicity rates. For example, with 6 patients, the probability of failing to observe toxicity occurring at least 40% of the time is less than 5%.

**Table 2:** Probability of escalation to the next higher dose for each underlying true DLT rate.

True underlying DLT	5%	10%	20%	30%	40%	50%	60%	70%	80%	90%
Probabilty of escalating dose	0.97	0.91	0.71	0.49	0.31	0.17	.08	0.03	0.01	0.001
Probability of Failing to Observe toxicity if sample = 3	0.86	0.73	0.51	0.34	0.22	0.13	0.064	0.027	0.008	0.001
Probability of Failing to Observe toxicity if sample = 6	0.74	0.53 0.26	0.12	0.047	0.016	0.0041	<0.001	<0.001	<0.001	<0.001

### Treatment and study assessments

Patients received oral pazopanib once daily at a dose of 800mg for 4 weeks before the administration of ^131^I. This run in period was conducted to establish the toxicities of pazopanib alone. During this period a dose reduction to 600 mg once daily was allowed if grade ≥ 3 adverse events were encountered. After the run-in period, the pazopanib was continued and ^131^I treatment planning and treatment were initiated.

For radioiodine treatment planning, patients underwent a minimum 1 week low- iodine diet, which was continued through dosimetry, pre-therapy imaging until 3 days post ^131^I treatment. On the two days prior to diagnostic dosing for radioiodine dosimetry, patients received 0.9 mg recombinant human thyroid-stimulating hormone (rhTSH, Thyrogen^®^ [thyrotropin alfa for injection], Genzyme Corporation, Cambridge, MA, USA) administered intramuscularly (IM). This was followed by administration of 3 mCi ^131^I for imaging and dosimetry. Dosimetry procedures followed the method of Benua,[7] where radioiodine whole body (WB) and blood clearance was measured starting on the day of dose administration and continuing for a total of 5 days. Patients underwent whole-body scanning 48, 72, and 96 hours after dose administration, with iodine uptake judged primarily based upon the 48 and 72 hour scans. WB and blood clearance curves were used to estimate the dose to marrow (taken from the radiation dose to blood) in rads/mCi. The ^131^I radioactivity dose was then chosen to match the designated marrow dose level in the dose escalation study. As part of the dosimetry procedure, it was confirmed that the chosen dose was unlikely to result in lung toxicity by assuring that less than 80 mCi of ^131^I was retained in the WB at 48 hours. Pulmonary toxicity considerations were not found to limit iodine dosing in this iodine-refractory population. Two days following the completion of dosimetry, patients underwent two daily injections of rhTSH, followed by therapeutic ^131^I dosing. 5–10 days after the therapeutic ^131^I administration, a post-therapy radioiodine scan was performed to determine the uptake of ^131^I in tumor tissue.

Because of concerns for additional additive hematologic toxicity that may occur with the combination of ^131^I with pazopanib, the starting dose of ^131^I was 50 rads with subsequent cohorts planned until the clinically established single agent dose of 150 rads was reached. Pazopanib continued to be administered once daily for 8 weeks post radioiodine therapy, for a total of 12 weeks of therapy. Anatomic tumour imaging and suppressed thyroglobulin levels were then performed within a week of discontinuing pazopanib therapy to establish a post-treatment follow-up baseline. Post-therapy tumor response and TTP was determined with respect to post pazopanib therapy baseline imaging and suppressed serum thyroglobulin levels taken just before rhTSh administration for dosimetry. Progression was determined by clinical radiologic progression by RECIST 1.1 criteria comparing baseline measurements to either radiologic imaging after pazopanib termination, increase in suppressed serum thyroglobulin levels by >50% compared to post-pazopanib levels, or both.

### Assessment of impact on tumor Iodine uptake

The impact of pazopanib on iodine uptake was assessed by comparing the post-pazopanib pre- and post-therapy radioiodine scans for each patient to historical pre- and post-therapy treatment scans performed prior to the pazopanib/^131^I therapy dose. Each patient was then judged to be in one of the following categories: 1) minimal or absent tumor iodine uptake or 2) moderate iodine or better uptake based on visualization of radioiodine above background levels in sites of known disease on anatomic imaging and/or FDG PET/CT. In addition, a qualitative estimate of any change in tumor iodine uptake with pazopanib was noted. The assessment was most heavily weighted on the post-therapy scans, where the best visualization of iodine uptake is expected. Radioiodine image reviewers were blinded to the patient outcomes.

### Correlative FDG-PET imaging

As per clinical routine staging for iodine-refractory patients at our center, patients underwent FDG PET/CT within 1 week of pazopanib initiation. As part of the study, patients underwent a second FDG-PET within 1 week prior to ^131^I administration as part of an exploratory assessment of the impact of pazopanib on the tumor, based upon our prior experience with other TKIs applied to iodine-refractory thyroid cancer.[18] The studies were compared using RECIST response criteria version 1.1.[19]

### Statistical methods

The primary end point of this phase I study was to assess the toxicity and the occurrence of dose limiting toxicity (DLT) of pazopanib given in conjunction with radioiodine in order to define the maximum tolerated dose (MTD)/recommended phase II dose (RP2D) in patients with RAI-refractory disease with minor RAI- uptake. We followed a traditional 3+3 cohort escalation design where for each cohort 3 patients were enrolled. DLT was defined as any study-drug related adverse event that occurred during the RAI therapy through to the end of pazopanib therapy at 8 weeks post RAI and was either at least a grade 3 according to the common terminology criteria for adverse events V.4.0 (except for diarrhea, nausea or vomiting)., grade 3 hematologic toxicity that lasted more than 5 days, grade 4 hematologic toxicity, febrile neutropenia or any treatment delay due to toxicity that lasted more than 14 days since the last dose of pazopanib. The MTD/RP2D was defined as the highest dose level at which 0 of the 3 patients or 1 of the 6 patients experience a DLT. The study population for toxicity analyses included all patients enrolled on study who received at least 1 dose of pazopanib. Efficacy was determined based on patients who received the combination of pazopanib and ^131^I treatment. Due to the exploratory nature of this study, no confirmatory inferential analyses were planned, and no imputation for missing data was performed. Descriptive statistics (e.g., means, medians, standard deviations, ranges) were used to summarize patient characteristics, treatment administration and compliance, efficacy, safety, and correlative study data.

Median progression-free survival (PFS) was estimated using the Kaplan–Meier method. PFS was timed from enrollment to death or progression (whichever was first). At the end of the study, patients without evidence of progression or death were censored at the date of last radiographic assessment of progression. Microsoft Office Excel was used for calculations.

## Results

### Patient characteristics

**Fig 1:**
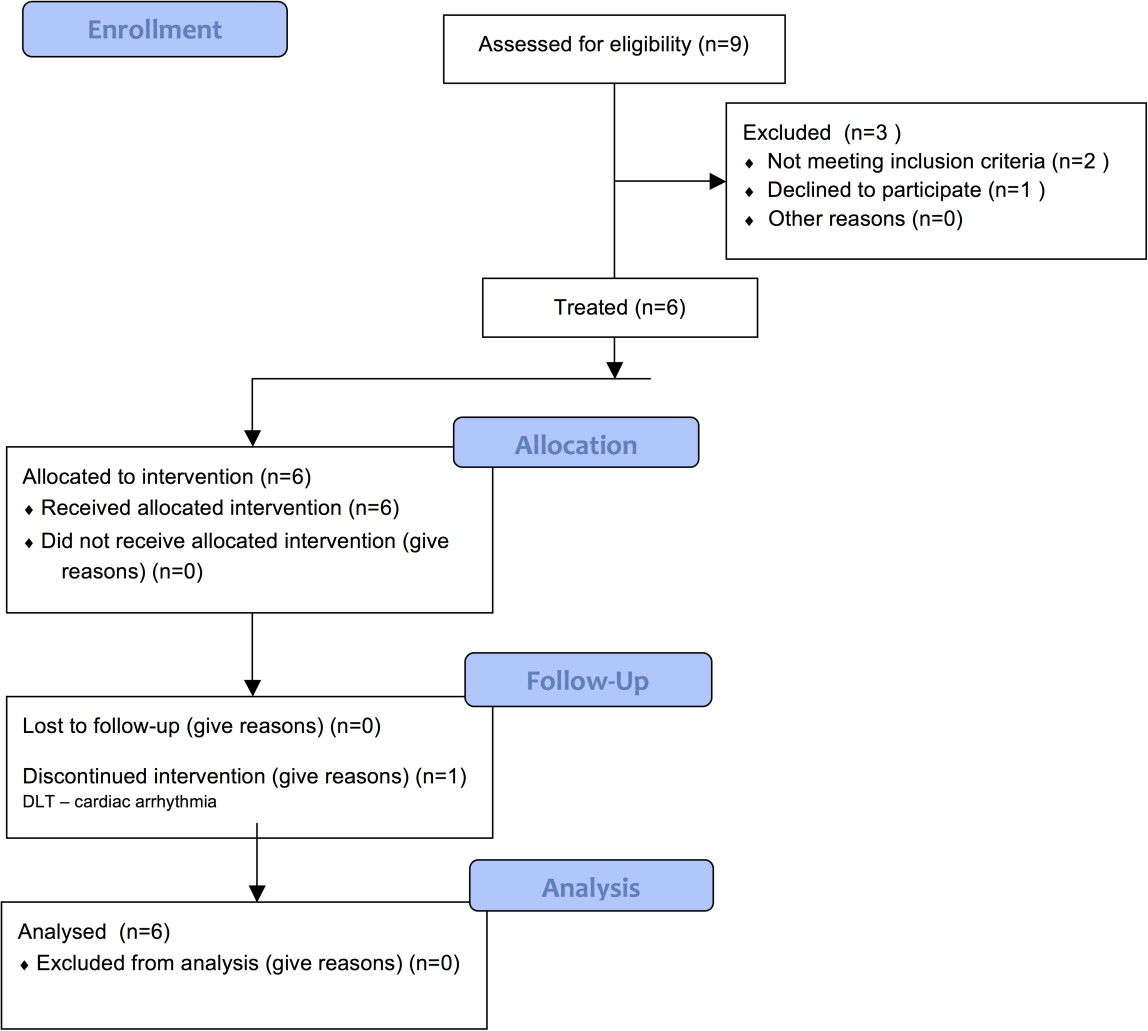
Consort flow diagram.

From December of 2011 to March of 2014 a total of 6 patients with WDTC participated in the study (Fig 1); baseline characteristics are summarized in Table 3. A papillary histology was present in 3 patients, one had follicular histology and another had a papillary-follicular variant. The protocol was amended to allow a patient with a thyroid-type carcinoma arising in Struma ovarii, a monodermal variant of ovarian teratoma that is composed of thyroid tissue has the typical gene rearrangements found in many cases of WDTC and also responds to ^131^I.[20] The median time from initial diagnosis of metastatic disease was 49.5 months (range 5–80 months) and median time from last ^131^I administration was 18.5 months (range 5–80 months).

**Table 3:** Patient demographics and baseline characteristics.

Age	Gender	ECOG PS	Histology	Criteria for study participation	Number of prior ^131^ therapies.*	Prior Cumulative I^131^ dose	Time since last I^131^ therapy (Months)	Times since diagnosis of metastatic disease (months)	Metastatic sites	PFS
72	Male	0	Follicular	3	3	930	23	78	Lung, Bone	107
37	Female	0	Papillary arising in a Struma ovarii	4	3	757	14	50	Liver, retroperitoneal lymphadenopathy	147
58	Male	0	Papillary-follicular	4	2	290	6	18	Bone	433
54	Male	0	Papillary	3	1	154	49	49	Lung, bone	202
65	Female	1	Papillary	1,2	1	158	5	5	Lung	548
59	Male	1	Papillary	1	2	Unknown	80	80	Bone, Mediastinal mass	253

*. Criteria to participate in the study 1) One or more measurable lesions with low or absent ^131^I uptake on the most recent pre-study radioiodine scans. 2) Disease progression according to RECIST criteria in imaging studies within 12 months of ^131^I therapy despite ^131^I uptake on RAI scan. 3) >50% increase in suppressed thyroglobulin levels during 12 months of I-131 therapy despite ^131^I uptake on RAI scan). 4) Evidence of at least one site of known disease with preserved ^131^I uptake above background levels on a diagnostic post-therapy ^131^I scan prior to study entry.

### Safety

During the pazopanib only phase of treatment, a patient required a dose reduction for fatigue and general malaise. During the first cohort of ^131^I escalation, 38 days after ^131^I administration, the same patient developed severe fatigue, chest pain, shortness of breath, palpitations and an irregular heartbeat. The ECG revealed that on top of an underlying rhythm of atrial fibrillation there were ST-T wave changes, as well as bigeminy with frequent premature ventricular contractions and ventricular pauses. Pazopanib was held due the perceived risk while awaiting cardiac work up and evaluation and the severe drug-related fatigue for more than 2 weeks. Although the palpitations and ST changes resolved and the patient was not felt to be at risk of a cardiac event by cardiology, the combined adverse events necessitating a treatment hold of greater than two week met criteria to be considered a dose limiting toxicity necessitating that the cohort expand to 6 patients. Other grade 2–3 adverse events attributable to radioactive iodine and pazopanib in combination are summarized in Table 4. Because of thrombocytopenia one patient had to have his pazopanib dose reduced 19 days after the administration of ^131^I. The combined administration of pazopanib and I^131^ appeared to have increased additive toxicities–particularly those of fatigue, and hematologic toxicities. Serious AEs (SAEs; ie, grades 3–4) occurred in 3 of the 6 patients of patients and included neutropenia, thrombocytopenia, lymphopenia and fatigue (Table 4). There were no patient deaths during the study.

**Table 4:** Adverse events related to treatment (pazopanib +/- I^131^) observed in patients on study (*N* = 6)^*^.

Adverse Event	All Grades	Grade 1–2	Grade 3–4
Fatigue	6 (100%)	5 (83%)	1 (17%)
Anorexia	5 (83%)	5 (83%)	
Diarrhea	4 (67%)	4 (67%)	
Dysgeusia	4 (67%)	4 (67%)	
Nausea	3 (50%)	3 (50%)	
Transaminemia	3 (50%)	3 (50%)	
Epistaxis—intermittent nose bleeds	2 (33%)	2 (33%)	
Hair color change	2 (33%)	2 (33%)	
Hypertension	2 (33%)	1 (17%)	1 (17%)
Palmar-plantar erythrodysesthesia syndrome	2 (33%)	2 (33%)	
Thrombocytopenia	2 (33%)	1 (17%)	1 (17%)
Alopecia	1 (17%)	1 (17%)	
Anal pain	1 (17%)	1 (17%)	
Anhedonia	1 (17%)	1 (17%)	
Arthralgia	1 (17%)	1 (17%)	
Hyperbilirrubinemia	1 (17%)	1 (17%)	
Depression	1 (17%)	1 (17%)	
Dry skin	1 (17%)	1 (17%)	
Gastroesophageal reflux disease	1 (17%)	1 (17%)	
Intestinal cramping	1 (17%)	1 (17%)	
Headache	1 (17%)	1 (17%)	
Lymphopenia	1 (17%)		1 (17%)
Insomnia	1 (17%)	1 (17%)	
Malaise	1 (17%)	1 (17%)	
Memory impairment	1 (17%)	1 (17%)	
Nail changes	1 (17%)	1 (17%)	
Nasal pain	1 (17%)	1 (17%)	
Neutropenia	1 (17%)		1 (17%)
Substernal chest tightness	1 (17%)	1 (17%)	
Sweating	1 (17%)	1 (17%)	
Ventricular arrhythmia	1 (17%)	1 (17%)	
Weight loss	1 (17%)	1 (17%)	

*toxicities reported were considered by the investigators as being related to pazopanib primarily with possible additive contributing I^131^radioactive iodine effects

### Antitumor activity

**Fig 2:**
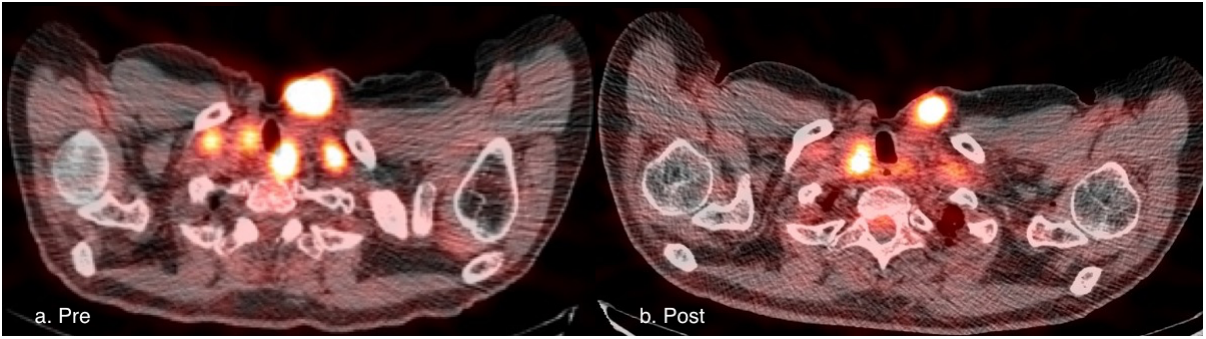
Fusion PET/CT image at baseline (A.Pre) and after 4 weeks of therapy with pazopanib (B.Post) in a patient with well-differentiated thyroid cancer. Post-pazopanib scan demonstrate some improvement in FDG-avid disease, although overall this patient had stable disease (Patient 6). FDG, ^18^F-fluorodeoxyglucose; PET, positron-emission tomography.

**Fig 3:**
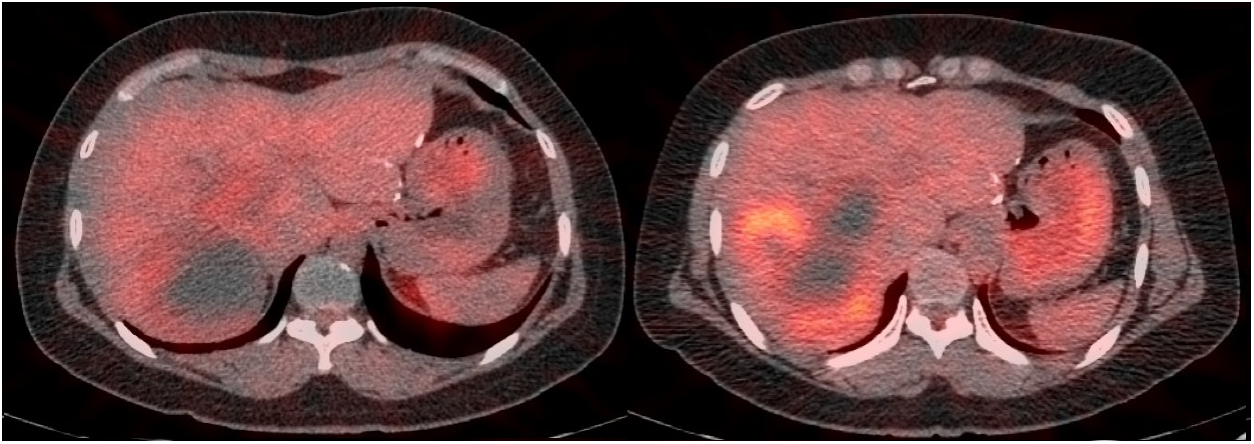
Fusion PET/CT scans at baseline (A.Pre) and after 4 weeks of therapy with pazopanib in a patient with well-differentiated thyroid. Post-pazopanib scan (B.Post) demonstrates new disease along superomedial margin of RFA site (Patient 2). RFA, Radiofrequency ablation; PET, positron-emission tomography.

**Fig 4:**
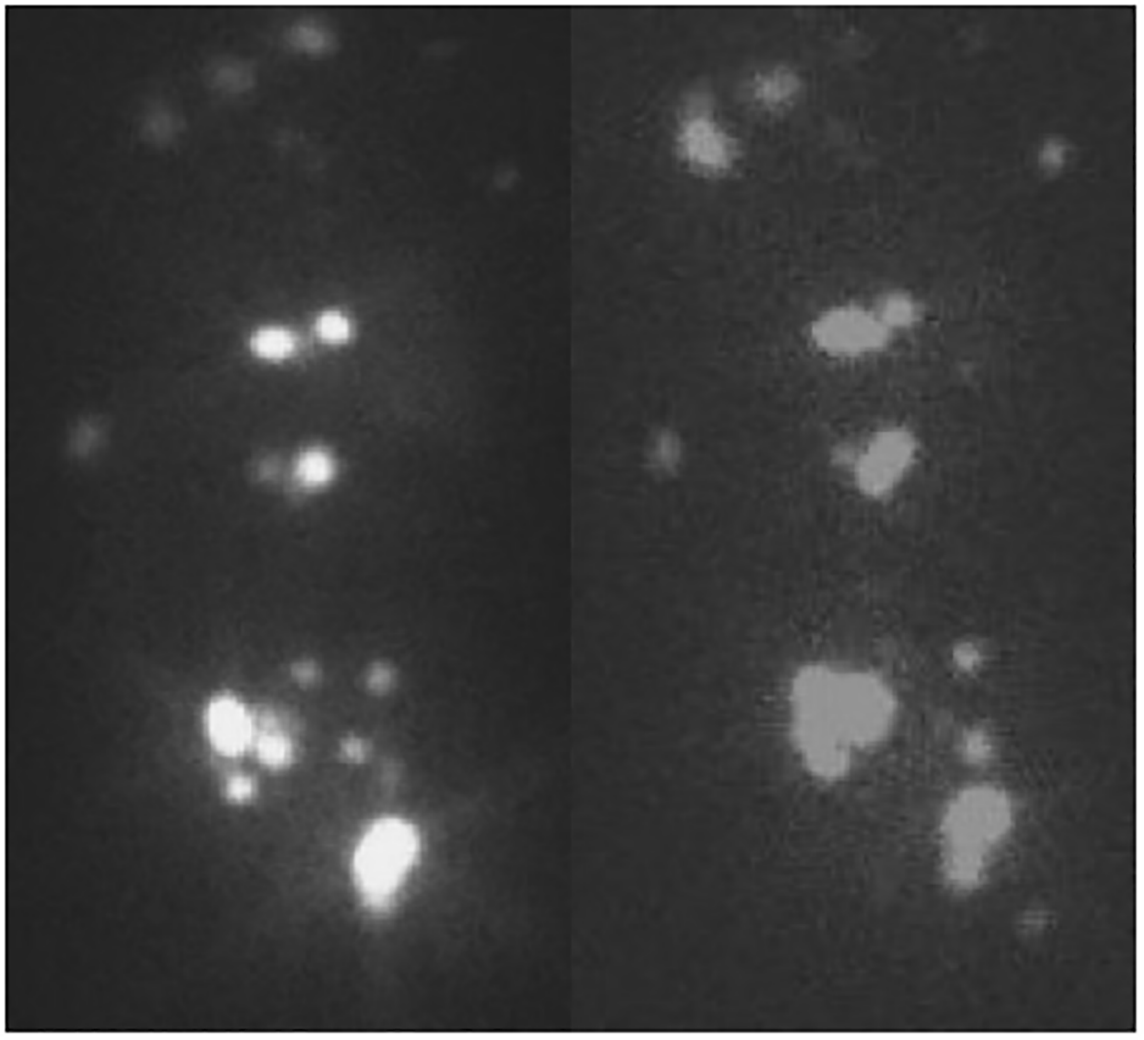
Historical (left) and post-pazopanib (right) post-^131^Iodine therapy images demonstrate multiple metastatic lesions, without increase in iodine-avidity post pazopanib.

A PET scan, obtained after the initial 4 weeks of pazopanib and before the initiation of ^131^I, was compared to a baseline scan to measure changed in FDG uptake. All 6 patients had one or more FDG-avid lesions on pre-pazopanib PET. Five patients had stable metabolic disease and one patient had new metabolically active disease indicative of metabolic progression (Figs 2 and 3). No patient had a significant increase in lesion iodine uptake with pazopanib (Fig 3). No significant increase in iodine uptake in lesions after pazopanib treatment was found (Fig 4).

Restaging imaging studies at the end of ^131^I treatment demonstrated that in the five patients with measurable lesions, four had stable disease and one had progressive disease defined as the appearance of new lesions. Thyroglobulin response could be evaluated in four patients (one had known anti-thyroglobulin antibodies and another was not compliant with bloodwork after ^131^I treatment). Three patients had a decrease in thyroglobulin levels compared to baseline (43–94%) and an increase of 73% was found in another patient. Median progression free survival was 6.7 months. One patient died of progressive disease without receiving subsequent therapy 10 months after the trial ended; two patients have started subsequent therapies which included sorafenib 5 months after trial completion and radiation and sunitinib 13 months after trial completion. The last 3 patients have not received subsequent therapy and are being actively being followed 15, 27 and 35 months from the last dose of pazopanib.

Of the 6 patients included, 3 were judged to have minimal to absent iodine uptake on historical study prior to pazopanib, while 3 were judged to have at least moderate iodine uptake. No patient had a significant increase in lesion iodine uptake with pazopanib (Fig 3).

## Discussion

We hypothesized that the combination of pazopanib and ^131^I may be theoretically beneficial in iodine refractory disease because of their independent mechanisms of anti-tumor effect and possibly through the impact of pazopanib in enhancing the sensitivity to ^131^I by inhibiting pathways that impact iodine uptake and retention. Although pazopanib was well tolerated initially, a DLT was found in the first cohort of the escalating doses of ^131^I at a 59 rad marrow dose and 83.9 mCi of ^131^I, no further DLTs were seen when the cohort was expanded, and thus the MTD/RP2D was found. Because of slow accrual to the study and the lack of lack of clear evidence of synergy between pazopanib and ^131^I, the combination was not pursued further. Despite the relatively low dose of radioiodine reached in the study (50 rad to the marrow, 83.9–251 miCi ^131^I) the regimen was active with a reduction of thyroglobulin seen in 2 of the 4 evaluable patients, and disease stability in several patients that had previously progressed. Unfortunately, these responses were short lived in 3 patients but ongoing in another 3 who have not required further therapy.

Other strategies to re-induce radioiodine sensitivity in thyroid cancer have included retinoids, thiazolidinediones, lithium, histone deacetylase inhibitors and other TKIs. Results have been modest at best in most prior attempts, with no real improvement or evidence of impact on iodine uptake or response until recently. In our study, there was no convincing evidence that the administration of pazopanib improved iodine uptake or retention and futher studies using this approach should not be perfomed. We note that encouraging results in restoring radioiodine sensitivity were found with inhibition of the MAPK pathway with selumetinib and debrafenib,[21] and studies investigating this strategy are ongoing.

Our group previously found that in patients with WDTC treated with sunitinib, a decline of 20% in FDG-activity early in treatment was somewhat predictive of clinical benefit to treatment (response or stable disease), and that patients with an increase in FDG uptake were likely to have progression.[18] In our current study, the small sample size does not permit us to draw any conclusions on the value of FDG PET/CT for assessing pazopanib response.

## Conclusion

Despite some apparent anti-tumor activity, the combination of pazopanib and ^131^I had unacceptable toxicity. This finding, together with a lack of apparent synergy with ^131^I, discourages trials of this therapy combination.

## Supporting information

S1 File Protocol that was IRB approved and followed for this study. (PDF)

S1 Fig TREND statement checklist. (PDF)
