# Supplementary material for: A phase I study of pazopanib in combination with escalating doses of 131I in patients with well-differentiated thyroid carcinoma borderline refractory to radioiodine
Source: PLoS One. 2017 Jun 29;12(6):e0178325. doi: 10.1371/journal.pone.0178325 (PMC5490965; doi:10.1371/journal.pone.0178325)
Supplement: S1 File — (PDF) [file pone.0178325.s001.pdf]

IRB Approved
Document Release Date: 10/22/2015

7529

University of Washington/ Seattle Cancer Care Alliance/
Fred Hutchinson Cancer Research Center
Current Version: 08/06/2013
Previous Version: 07/02/2012

Clinical Study Protocol

Title: A Phase I Clinical Trial of Pazopanib in Combination with Escalating Doses of Radioactive ^{131}I in Patients with Well-Differentiated Thyroid Carcinoma Refractory to Radioiodine, Despite Having Some Uptake

Principal NCCN Investigator: Laura QM Chow[1]

The University of Washington, MS G4-940, Division of Medical Oncology (box 358081) , 825 Eastlake Avenue East, , Seattle WA, 98109-1023
Phone: 2062886968, Fax: 2062887543
Email: lchow@seattlecca.org

Co-Investigators: David Mankoff,[1] Renato Martins[1], Keith Eaton[1], Richard Alan Failor[1], Robert Doot,[1] and Brenda Kurland.[2]

[1]University of Washington and the Seattle Cancer Care Alliance, Seattle, WA, USA
[2] Fred Hutchison Cancer Research Center and the Seattle Cancer Care Alliance, Seattle, WA, USA

TABLE OF CONTENTS

1.0	SUMMARY…………………………………………………………………......	4
2.0	BACKGROUND………………………………………………………………..	5
3.0	STUDY POPULATION AND SELECTION OF SUBJECTS…………………………	14
4.0	TRIAL DESIGN AND TREATMENT PLAN……………………………………………	18
5.0	STUDY CONDUCT AND TRIAL PROCEDURES……………………………………	25
6.0	DATA ANALYSIS/STATISTICAL METHODS………………………………………...	40
7.0	ASSESSMENTS………………………………………………………………………	42
8.0	REGULATORY, QUALITY AND ADMINISTRATIVE REQUIREMENTS…………..	47
9.0	REFERENCES………………………………………………………………………	49
10.0	SUPPLEMENT AND APPENDICES……………………………………………………	53

LIST OF TABLES

Table 1	Definitions for Adequate Organ Function…………………………………	15
Table 2:	Dose Levels for Pazopanib in Combination with RAI................................	20
Table 3:	Dose Modification Algorithms for Potential Treatment-Related Adverse Events………………………………………………………………………	35
Table 4:	Guidelines for Management of Treatment Emergent Hepatotoxicity……...	39
Table 5:	Probability of Escalation to the Next Dose for Each True Underlying DLT Rate at a Dose Level………………………………………………………...	40
Table 6:	Probability of Failing to Observe True Underlying DLT Rate at a Dose Level…………………………………………………………………………	41
Table 7:	Response Evaluation Criteria in Solid Tumors……………………............	60

LIST OF FIGURES

Figure 1: Treatment Schema………………………………………………………...24

1.0 SUMMARY

Title

A Phase I Clinical Trial of Pazopanib in Combination with Escalating Doses of Radioactive ^{131}I in Patients with Well-Differentiated Thyroid Carcinoma Refractory to Radioiodine, Despite Having Some Uptake

Indication: Recurrent or metastatic well-differentiated thyroid carcinoma (WDTC) patients with radioactive iodine (RAI)-avidity but progressive disease after high-dose RAI, or in RAI-refractory patients who still maintain some RAI uptake in one or more sites of known disease.

Hypothesis:

The combined actions of the multi-targeted anti-angiogenic tyrosine kinase inhibitor (TKI), pazopanib, and concurrent ^{131}I radioiodine will have additively or synergistically overcome therapeutic ^{131}I resistance in recurrent or metastatic well-differentiated thyroid carcinoma (WDTC) patients with radioactive iodine (RAI)-avidity but progressive disease after high-dose RAI, or in RAI-refractory patients who still maintain some RAI uptake in one or more sites of known disease, by (1) improving delivery of ^{131}I in bulky tumors by vascular normalization, by (2) increasing sensitivity to the radiation effects, and/or by (3) independent, combined anti-tumor effects.

Objectives

Primary Objectives:

- To determine the safety, tolerability and feasibility of administrating escalating doses of ^{131}I in combination with concurrent pazopanib therapy in order to define the maximum tolerated dose (MTD)/recommended phase II dose (RP2D) in patients with RAI-refractory disease with minor RAI-uptake.

Secondary Objectives:

- To determine the effects of pazopanib in combination with ^{131}I on RAI-avidity, uptake and tumor response rate (RECIST version 1.1).
- To determine the time to tumor progression (TTP) or recurrence (progression will be determined by RECIST criteria and by increases in suppressed thyroglobulin levels >50% as compared to tumor imaging and suppressed thyroglobulin levels performed within 1 week of the last dose of pazopanib).

Research Clinical Trial Design:

This is a phase I open-label, dose-finding, safety study of escalating doses of ^{131}I RAI in combination with pazopanib in adult patients with advanced recurrent or metastatic WDTC that is RAI-refractory, but still maintains some minor RAI uptake at one or more known sites of disease. This will be a single center, un-controlled, non-comparative and non-randomized trial with no stratification.

Anticipated first patient in: September 2011, anticipated last patient out: December 2013
Duration of Treatment Period: 18 to 22 months recruitment and treatment, 6 months follow-up.
Number of patients**:** 12 -15 (estimated), maximum of 24 patients.
Estimated Annual Accrual: 9 -12 patients.
Expected date of Final report: May 2014

2.0 BACKGROUND

Although thyroid cancer only comprises 1-2% of all cancers, it is the most common endocrine malignancy.[1,2] Its incidence has doubled in the last decade and continues to rapidly rise, causing substantial morbidity and mortality: 44,670 estimated new cases and 1690 deaths due to thyroid cancer were recorded in the United States in 2010.[3,4] The major epidemiologic factors for thyroid cancer are radiation exposure and iodine deficiency. Localized well-differentiated thyroid carcinoma is treated well with surgical excision (near total/total thyroidectomy, or unilateral lobectomy with isthmusectomy) followed by radioiodine 131 ablation of the remnant.[5] Although many patients will be cured with the above approach, 10-15% of WDTC patients will develop recurrent and/or distant metastatic disease.[6] At least 5% of patients with advanced thyroid cancer develop life-threatening progressive disease with a dismal 10 year overall survival for WDTC of less than 40%.[2] WDTC is compromised of predominantly (> 90%) papillary and follicular subtypes which are generally radioactive iodine-avid.[7] When patients with advanced WDTC become iodine-refractory; however, there are no standard of care options and systemic chemotherapy is of little benefit.

Radioiodine ^{131}I therapy has been a highly effective mainstay of therapy since the 1940's with undisputable clinical benefits in recurrent or metastatic WDTC patients with high RAI-avidity and uptake.[8] Most RAI-avid WDTC patients respond well to ^{131}I therapy, except patients with bulkier tumors and predominant bone disease.[9,10] WDTC patients are considered refractory to RAI when their cancers no longer have any ^{131}I uptake, or when they fail to respond to ^{131}I RAI despite evidence of uptake.[11,12] Furthermore, patients with WDTC tumors with elevated glycolysis, demonstrated by ^{18}F-fluorodeoxyglucose (FDG) PET, are often resistant to RAI, even when their tumors are RAI-avid.[13] When patients become RAI-refractory and WDTC tissue fails to take up RAI, treatment options are limited and survival is greatly decreased: radiation therapy and traditional cytotoxic chemotherapy have failed to substantially impact survival.[7,8] The unmet need in this patient population has driven the search for more effective therapies. Due to the highly vascular nature of this tumor, many of the multi-targeted anti-angiogenic tyrosine inhibitors (TKIs), including sorafenib, axitinib, pazopanib, and particularly sunitinib in our own institutional phase II clinical study, have demonstrated significant promise and efficacy.[14-24] Although these agents had demonstrated substantial promise with tumor responses, stability, and improved time to progression (TTP) in the phase II setting in RAI-refractory patients, toxicities can limit chronic administration and responses may not be sustained.[14-24] Phase III clinical trials are ongoing to confirm the effectiveness and benefit of these agents in the advanced and metastatic setting.

Angiogenesis plays a prominent role in thyroid cancer: WDTC are highly vascular and microvessel density appears to correlate with disease-free survival.[25] The major angiogenic factor, the vascular endothelial growth factor (VEGF), is often overexpressed and VEGF receptor tyrosine kinases (RTKs) are frequently activated in WDTC and in the surrounding stoma. WDTCs with high VEGF expression may be associated with increased tumor size, local and distant metastastic spread.[26] Pazopanib, (GW786034, GSK, GlaxoSmithKline) is an oral, mutli-targeted tyrosine kinase inhibitor that has potent nanomolar in vitro activity against the vascular endothelial growth factor receptor (VEGFR), platelet-derived growth factor receptor (PDGFR), fibroblast Growth Factor Receptor (FGFR), c-Kit, c-fms and other key proteins involved in angiogenesis, cancer cell progression, growth and survival.[27] Potent anti-angiogenic and anti-tumor effects were observed with pazopanib in a number of in vivo xengraft models.[27] Pazopanib was well-tolerated in the phase I clinical trial setting with early clinical activity observed in renal cell carcinomas and pancreatic islet cell carcinoma. Importantly, evidence of pazopanib's anti-angiogenic effects were demonstrated with greater than 50% decreases in tumor blood flow on dynamic contrast-enhanced magnetic resonance imaging (DCE-MRI) in 10 out of 12 patients.[28] Pazopanib administered at a dose of 800 mg once daily in 4 week cycles in a phase II study of 37 patients with metastatic, rapidly progressive radioiodine-refractory differentiated thyroid cancer,

demonstrated exceptionally promising anti-tumor activity.[14] These patients had up to two prior previous therapies and had demonstrated radiographic progression in the 6 month period prior to enrollment. Treatment was well-tolerated and patients received a median of 12 cycles of therapy with confirmed partial responses in 18 patients (response rate of 49%, 95% CI 35-68) and a 66% likelihood of response lasting longer than a year.[14] The maximum concentration of pazopanib in plasma during cycle one appeared to correlate with radiographic response. The initial dose of 800 mg once daily continuous dosing appeared to be well tolerated; however, 43% of patients required dose reductions for fatigue, skin and hair hypopigmentation, diarrhea, and nausea.[14] Astonishingly, this study with pazopanib reported the highest partial response rate yet reported in patients with differentiated thyroid cancers and appeared to modify the disease course dramatically in responders.[14] The clinical activity of pazopanib seems to be better than any other VEGF receptor kinase inhibitor agent to date.[14] Due to this promising activity, pazopanib is being assessed further assessed in confirmatory phase III trials in well-differentiated (papillary and follicular), medullary and anaplastic thyroid carcinomas.

Although radioiodine ^{131}I therapy is highly effective, treatment can be limited by its side effects. Acute nausea, vomiting and dehydration are common but preventable and treatable; whereas, radiation thyroiditis, painless neck edema, sialadenitis and tumor hemorrhage or edema occur less commonly in 10-20% of patients – but are seen predominantly at higher doses.[29,30] Chronically, there is the risk of secondary malignancies such as bladder, salivary, gastrointestinal tract cancers and possibly breast cancer and acute myeloid leukemia.[29,30] Moreover, gonadal dysfunction, particularly ovarian failure in woman can occur and obstruction of the nasolacrimal duct causing excessive tearing. Many of the chronic risks increase with higher cumulative doses.[29,30] Although RAI therapy is indisputably effective, very few studies have attempted to increase the activity and effectiveness of RAI by combinational techniques; therefore, the toxicities of RAI therapy in combination are not well elucidated and should be approached very cautiously. This will be the first phase I dose-finding study of pazopanib at standard doses in combination with escalating doses of ^{131}I radioiodine.

2.1 Medical Rationale

Hypothesis of ^{131}I and Pazopanib in WDTC:

The combined actions of the multi-targeted anti-angiogenic tyrosine kinase inhibitor (TKI), pazopanib, and concurrent ^{131}I radioiodine may additively or synergistically overcome therapeutic ^{131}I resistance in recurrent or metastatic well-differentiated thyroid carcinoma (WDTC) patients with radioactive iodine (RAI)-avidity but progressive disease after high-dose RAI, or in RAI-refractory patients who still maintain some RAI uptake in one or more sites of known disease, by (1) improving delivery of ^{131}I in bulky tumors by vascular normalization, by (2) increasing sensitivity to the radiation effects, and/or by (3) independent, combined anti-tumor effects.

Rationale of ^{131}I and Pazopanib in Combination in WDTC

The effectiveness of ^{131}I is dependent on its uptake, accumulation, and retention, and radiation delivery to WDTC cells in RAI-avid disease. Anti-angiogenic agents are ideally combined with ^{131}I in WDTC as 1) they have independent anti-tumor effects,[22,24] 2) anti-angiogenic agents in combination with chemotherapy transiently normalize tumor vasculature, increase permeability, reduce interstitial pressure, facilitate drug diffusion and reduce intratumoral hypoxia to enhance drug delivery to the tumor mass,[31-33] and 3) anti-angiogenic agents combined with radiation therapy enhance radiation-induced apoptosis with improved anti-tumor effects by antagonizing radiation-induced increased hypoxia, normalizing tumor vasculature and increasing oxygenation.[34,35] Our protocol addresses patients that have failed to respond to ^{131}I RAI despite evidence of uptake, and also patients that are RAI refractory,

but still maintain minor ^{131}I uptake in at least one site of known disease. There is no clear consensus as to the best course of action for these patients and discrepancy as to when to start anti-angiogenic TKI therapy, as the rate of clinical progression can vary and change from slow indolent growth to rapid progressive disease. If ^{131}I therapy in RAI-refractory patients could be optimized in combination with anti-angiogenic TKIs such as pazopanib, the morbidity and mortality of patients with recurrent and metastatic WDTC could be greatly reduced. Furthermore, this combination would also potentially expand the current use of anti-angiogenic TKIs from largely RAI-negative patients to patients with RAI-avid metastatic disease – a substantially larger group of patients.

Our study differs from prior studies that have used agents to try to enhance the efficacy of RAI in iodine-negative patients: six weeks of bexarotene pre-treatment prior to RAI did increase RAI uptake in non-iodine-avid WDTC patients in all eight of the patients, but the study numbers were too small to determine much clinical benefit to subsequent radioiodine therapy.[36] Similarly, lithium increased ^{131}I retention, prolongation, and increased the ^{131}I dose to the thyroid remnant and metastatic sites in 6 out of 7 patients in one study, and 7 out of 12 patients in another study; but study numbers were too small to determine a significant clinical benefit.[37-39] A single arm phase II study of sorafenib given for 26 weeks to 32 patients with metastatic or locally advanced RAI-refractory WDTC prior to RAI imaging did not restore or re-induce RAI-uptake in non-RAI-avid WDTC; however, combined therapy was not attempted.[40] There is no data in regards to the concurrent treatment of radioiodine with a multi-targeted anti-angiogenic TKI agent in patients that are RAI-refractory who still maintain some level of RAI-uptake.

In this trial, we will test the hypothesis that the combined actions of pazopanib and ^{131}I will overcome therapeutic resistance in patients with RAI-avidity but progressive disease after high-dose RAI by (1) improving delivery of ^{131}I in bulky tumors by vascular normalization, by (2) increasing sensitivity to the radiation effects, and/or by (3) independent, combined anti-tumor effects. Additive or synergistic anti-tumor effects may potentially be seen in the population who still retain some minor ^{131}I uptake. We propose that concurrent administration of pazopanib and ^{131}I may improve ^{131}I anti-tumor efficacy in refractory disease, prolong the TTP, and improve the quality of life by deferring the need to initiate life-long systemic anti-angiogenic therapy and its associated toxicities. There is no published data regarding this combination as anti-angiogenic TKIs have never previously been combined with radioiodine. Therefore, the safety and dose of ^{131}I in this situation are not known, and combined toxicities may be additive. We propose a phase I dose- finding, safety and feasibility study of combined pazopanib and escalating doses of ^{131}I therapy, in patients with relatively RAI-refractory disease with still minor RAI uptake. The optimal treatment of RAI-refractory recurrent or metastatic WDTC patients which still maintain minor RAI uptake is a common unresolved therapeutic dilemma. This combination would be a novel approach with high scientific merit which would lend valuable knowledge to an area previously unexplored. Importantly, correlative imaging with dynamic FDG-PET will also look at tumor perfusion, flow dynamics, and PET response with pazopanib alone in WDTC, and its effect on RAI-uptake, tumor response and TTP.

<u>Hypothesis and Rationale for Correlative Dynamic FDG-PET Imaging</u>

Position emission tomography (PET) can be used to monitor blood flow, blood volume, and vascular permeability, and to assess tumor perfusion and drug effects in animals and patient studies. A variety of radiotracers can monitor blood flow within tumors such as radioactive forms of water labeled with ^{15}O.[41] Although not a direct measure of biological activity, ^{18}F-deoxyglucose (FDG)-PET is a validated marker to monitor cellular metabolisms and determine metabolic changes to therapy in most solid tumors.[42] Phase I and II clinical trials have increasingly used FDG-PET as an early indicator of an anti-tumor effect. PET scans demonstrate changes in vascular permeability, volume fraction or metabolism after therapy, and may potentially predict the clinical efficacy of anti-angiogenic agents.[42]

Preliminary institutional data has been published demonstrating the use of early FDG PET to indicate the response to sunitinib in RAI-refractory thyroid cancer patients,[16] and in using dynamic PET to asses both perfusion changes and metabolism change of breast cancer in response to chemotherapy.[41] In our prior study of sunitinib in iodine-refractory thyroid cancer, patients underwent FDG PET pre- and one-week post-starting sunitinib, and a 20% decline in uptake was taken as evidence of a tumor response based upon prior published data for repeatability in PET [43,44] and our clinical experience. A decline of 20% in FDG early in treatment was somewhat predictive of clinic benefit to treatment (response or stable disease), and that patients with an increase in FDG uptake were likely to have progression. Dynamic FDG-PET can also be used to provide a more sensitive measure of response as well as an indicator of perfusion.[45] Doot RK et al. showed that dynamic PET provided up to 35% greater sensitivity for measuring declines in FDG uptake compared to static measures such as SUV.[45] Tseng J et al. showed measures of FDG delivery (typically called K_1) provided an indirect measure of tumor perfusion that correlated with validated measures of tumor blood flow.[41] Moreover, Tseng and Dunnwald showed that changes in FDG K1 were highly predictive of tumor response and outcome.[41,46] Frequently, dynamic FDG PET studies of cancer response are used at our institution and currently this method is being used in a number of projects, including an assessment of perfusion and metabolism for locally advanced breast cancer treated with combined sunitinib/chemotherapy. The additional dynamic FDG-PET studies and data will certainly add additional information regarding the activity and response of pazopanib in advanced WDTC patients.

2.1.1 Drug Information and Formulation

Please refer to the GlaxoSmithKline Pazopanib (GW786034) Investigator Brochure which gives additional information.[47]

Pazopanib (GW786034B) Tablets

GW786034B Tablets are supplied as 50 mg, 100 mg, 200 mg, 400 mg and 500 mg (as free base) tablets for oral administration to support oncology indications.[47] The 50 mg and 100 mg tablets are round, the 200 mg and 400 mg tablets are oval shaped or capsule-shaped, and the 500 mg tablets are capsule shaped. The 50 mg, 100 mg, and 500 mg tablets are white to slightly colored, while the oval-shaped 200 mg and 400 mg tablets are white. Additionally, the capsule-shaped 200 mg tablets can be gray or pink and may be debossed, and the capsule-shaped 400 mg tablets can be white or yellow and may be debossed. Tablets are packaged in white high density polyethylene (HDPE) bottles with white plastic, induction seal, child-resistant caps. [47]

7529

GlaxoSmithKline Laboratory Code:		GW786034B<br>The 'B' suffix denotes the monohydrochloride salt.
Approved Names	:	Pazopanib hydrochloride (USAN)<br>Pazopanib (INN)
Chemical Name	:	5-[[4-[(2,3-Dimethyl-2H-indazol-6-yl)methylamino]-2-pyrimidinyl]amino]-2-methylbenzenesulfonamide monohydrochloride
Structural Formula	:	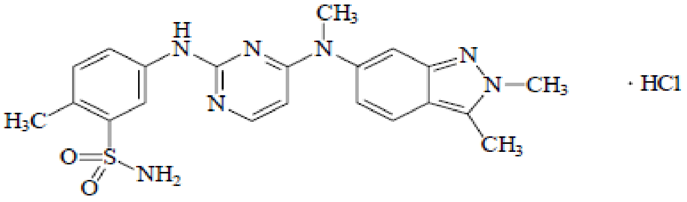
Molecular Formula	:	$C_{21}H_{23}N_7O_2S \bullet HCl$
Molecular Weight	:	473.99 g/mol (monohydrochloride salt)<br>437.53 g/mol (free base)
Physical Form	:	White to slightly colored solid.
Solubility	:	GW786034B is very slightly soluble in 0.1 M HCl (0.65 mg/mL), and is practically insoluble in pH 7.0 phosphate buffer (0.00005 mg/mL), and in pH 11 piperidine buffer (0.0002 mg/mL).

<u>**Summary of Pazopanib Non-Clinical Findings of Relevance to the Study**</u>

Pazopanib is an orally-bioavailable, adenosine tri-phosphate (ATP)-competitive tyrosine kinase inhibitor (TKI) of VEGFR (-1, -2, and -3), PDGFR (-α and -β), and c-Kit.[48,49] Pazopanib potently inhibited VEGFR-1, -2, -3, PDGFR-α and -β and c-kit, with half-maximal inhibition (IC50) values of 10, 30, 47, 71, 84 and 74 nM, respectively. [47,48] Pazopanib has demonstrated encouraging potency and selectivity for VEGF receptors: for example, pazopanib demonstrated significant inhibition of VEGF-induced VEGFR-2 phosphorylation in human umbilical vein endothelial cells as well as in mouse lungs in a dose dependent manner, and was 3- to 400-fold selective for VEGF receptors compared to 23 other kinases tested.

Effects on cell proliferation

The ability of pazopanib to inhibit proliferation of various cell types was evaluated in an in vitro assay for 3 days. Pazopanib selectively inhibited the proliferation of human umbilical vein endothelial cells (HUVEC) stimulated with VEGF (IC50 = 21 nM) compared to basic fibroblast growth factor (bFGF) stimulated proliferation (IC50 =721 nM). Pazopanib was further evaluated in a cell proliferation assay using a panel of 282 human cell lines. Only 7 tumor cell lines showed an IC50 value of <1000 nM, suggesting that pazopanib is a weak or inactive inhibitor of proliferation in the majority of human cell

lines tested in vitro. Since pazopanib inhibits c-Kit and Flt-3 receptors, which are expressed on hematopoietic progenitor cells and have a pleiotropic role during progenitor cell proliferation and differentiation of various hematopoietic lineages, the effects of pazopanib on bone marrow progenitor growth were investigated in multiple growth factor formats in standard colony forming assays in vitro. Pazopanib had weak activity in the colony forming unit assay induced by granulocyte-macrophage colony stimulating factor (GMCSF) and Flt-3 ligand alone. However, addition of stem cell factor (ligand for c-Kit) enhanced the ability of pazopanib to inhibit colony formation, consistent with its activity against c-Kit kinase. One of the circulating metabolites of pazopanib, GSK1268997, inhibited VEGF-induced endothelial cell proliferation with similar potency to that of pazopanib. The other 3 circulating metabolites, GSK1268992, GSK1071306 and GW700201, showed at least 10 to 20-fold less activity than pazopanib.

In summary, pazopanib potently inhibits VEGFR-1, VEGFR-2, VEGFR-3, PDGFR-α, PDGFR-β and c-Kit kinases. Consistent with its kinase activity, pazopanib selectively inhibited proliferation of endothelial cells stimulated with VEGF compared to bFGF stimulated proliferation and it had no direct anti-proliferative effect against the majority of the large number of tumor cell lines evaluated.

Inhibition of VEGFR2 phosphorylation

In vivo effects of pazopanib on VEGFR-2 phosphorylation, angiogenesis and tumor growth have been investigated in a variety of animal models. Inhibition of VEGFR-2 phosphorylation was studied in naïve mice given an intravenous bolus administration of VEGF. The lungs of mice that received VEGF showed increased phosphorylation of VEGFR-2 compared to untreated control mice. Pre-treatment of mice with a single oral dose of pazopanib inhibited VEGF-induced VEGFR-2 phosphorylation in lungs in a dose- and time-dependent manner. The results from the time course and dose-response experiments together suggest that plasma concentrations of ~40 μM or higher are required for the optimal inhibition of VEGFR-2 phosphorylation in mice.

Inhibition of angiogenesis

Pazopanib given orally at ≥ 30 mg/kg inhibited bFGF- and VEGF-induced angiogenesis in a variety of animal models including the Matrigel plug and corneal micropocket models of angiogenesis in Swiss nu/nu or C57B1/6 mice. Pazopanib also showed generally dose-dependent inhibition of aberrant ocular angiogenesis in laser-induced choroidal neovascularization in C57B1/6J mice (≥ 8 mg/kg PO) and Brown Norway rats (2.25 mg/kg, eye drops) as well as corneal neovascularization in a suture-induced model in New Zealand white rabbits (≥ 0.3 mg/kg, eye drops). These results are consistent with the role of VEGFR and PDGFR signaling in angiogenesis and demonstrate the effectiveness of pazopanib in blocking angiogenesis induced by various approaches.

Anti-tumor activity in human tumor xenografts in mice

Pazopanib showed significant growth inhibition of a variety of human tumor xenografts in mice, and also inhibited basic fibroblast growth factor (bFGF) and VEGF-induced angiogenesis in several different models of angiogenesis (e.g., the Matrigel plug assay, and the cornea micropocket and laser-induced choroidal neovascularization models).[47-49] The anti-tumor activity of oral pazopanib has been investigated as a single agent and in combination with various antineoplastic agents in various human tumor xenograft models in mice. Pazopanib was administered orally in these studies on a once or twice daily schedule and was well tolerated. Pazopanib inhibited the growth of different tumor xenografts to varying degrees. Pazopanib had no effect on the growth of prostate tumors in the transgenic prostate cancer mouse model, CR2-T-Ag. These data demonstrate that different tumors have varying dependence on angiogenesis and VEGFR/PDGFR signaling. Since treatment with pazopanib alone does not eradicate tumors in mouse models, the effect of combining pazopanib with various antineoplastic agents was evaluated, including various signal transduction inhibitors using human tumor

xenograft models in mice. In general, the combination of pazopanib with all agents was well tolerated in mice in these studies, with no significant increase in body weight loss or any overt clinical effects.

Pazopanib Clinical Safety Summary

Approximately 5390 subjects with cancer have been enrolled in clinical studies of pazopanib (including studies conducted by the National Cancer Institute [NCI], part of the United States National Institutes of Health) as of 09 September 2010. Data collected to date show that oral pazopanib is absorbed after administration and that pazopanib administration at 800 mg daily is associated with a reasonable safety profile and encouraging efficacy in various oncology settings.

In a renal cell carcinoma (RCC) study where pazopanib was compared to placebo, the median time on treatment was approximately twice that on placebo (7.4 months versus 3.8 months). The overall frequency of adverse events (AEs) reported during the study was higher in the pazopanib arm (92%) compared with placebo (74%).[47,50,51] Most common AEs reported in >20% subjects in the pazopanib arm (as of 23 May 2008) were diarrhea (52%), hypertension (40%), hair color change (depigmentation; 38%), nausea (26%), anorexia (22%), and vomiting (21%). These AEs were all reported at a higher incidence in the pazopanib arm than in the placebo arm. Most of these events were Grade 1 or 2 using the National Cancer Institute Common Toxicity Criteria for Adverse Events (NCI CTCAE) Version 3.0. More Grade 3 AEs were reported in the pazopanib arm (33%) compared with the placebo arm (14%). The frequency of Grade 4 AE and Grade 5 events was similar between the pazopanib and placebo arms: Grade 4 in 7% and 6%, respectively; Grade 5 in 4% and 3%, respectively. At the time of the final analysis, a subsequent review of safety data did not reveal any changes to the previously observed safety profile; no new safety signals were detected.

Based on the analysis of the safety data integrated across three RCC studies, as of 09 January 2009 (N=593, the most common AEs and serious adverse events (SAEs) were similar to those observed in the pazopanib arm.[47,50,51] Rare but serious AEs previously described for VEGFR inhibitors, such as cardiac/cerebral ischemia, hemorrhage, and bowel perforation, were observed with pazopanib treatment. A review of SAEs across oncology studies revealed that the most frequently reported SAEs (≥ 50 events), regardless of causality and treatment regimen, as of 09 September 2010 in decreasing order of frequency were alanine aminotransferase (ALT) increased, vomiting, dyspnea, abdominal pain, diarrhea, dehydration, pyrexia, fatigue, pneumonia, anaemia, aspartate aminotransferase (AST) increased, nausea, pleural effusion, hypertension, and pulmonary embolism. The most common chemistry abnormalities, occurring almost twice as frequently on pazopanib compared with placebo included ALT (53% versus 22%), AST (52% versus 19%) and bilirubin elevations (36% versus 10%), hypophosphatemia (34% versus 11%), hypomagnesemia (26% versus 14%), hypoglycemia (17% versus 3%) and hypokalemia (9% versus 2%). Most of these abnormalities were Grade 1/2. The most common Grade 3/4 abnormalities were ALT and AST elevations. Although leukopenia, neutropenia, and thrombocytopenia were more common on pazopanib than placebo, Grade 3/4 cytopenias were uncommon. For pazopanib, the major laboratory abnormality appears to be elevation of hepatic enzymes, which typically occurred during the first 18 weeks of treatment. As of 09 January 2009, the transaminase elevations were reversible in 96 (91%) of 106 subjects with elevated ALT ≥ 3x upper limit of normal (ULN); 7 of the remaining 10 subjects had limited or no follow-up to determine recovery and 3 died of cancer progression with no follow-up ALT data. It was noted early in development that some of the subjects with elevated hepatic enzymes remained on study drug despite these elevations and had normalization of their transaminases while remaining on pazopanib ("adaptation"). Most subjects with transaminase elevations in whom dosing was interrupted could be successfully re-challenged.[47,50,51]

In the over 5000 patients enrolled in pazopanib clinical studies as of September 2010, approximately 2974 serious adverse events (SAEs) were reported across all pazopanib oncology clinical trials.[47] This estimate includes SAEs from GSK sponsored and supported studies, and both placebo, combination

therapy, and comparator data. A review of SAEs across oncology studies revealed that the most frequently reported SAEs (≥ 50 events), regardless of causality and treatment regimen, as of 09 September 2010 in decreasing order of frequency were: alanine aminotransferase increases, vomiting, dyspnoea, abdominal pain, diarrhea, dehydration, pyrexia, fatigue, pneumonia, anaemia, aspartate aminotransferase increases, nausea, pleural effusion, hypertension, and pulmonary embolism. Overall, 33% (990/2974) of the SAEs reported were assessed as related to the Investigational Product by the Investigator. The majority of the SAEs of dehydration occurred in association with events of vomiting, diarrhea, and/or decreased oral intake. This highlights the importance of proactive supportive care in patients with diarrhea, vomiting or decreased oral intake.[47]

Pazopanib received first marketing authorization in the United States on 19 October 2009, and currently is approved in a number of countries for use in advanced and/or metastatic renal cell carcinoma. As of 09 September 2010, GSK has received approximately 562 post-marketing spontaneous adverse events (serious and non serious). These include events reported by health care professionals and consumers. The most frequently reported events from marketed use include fatigue, diarrhea, nausea, decreased appetite, death, hair color changes, hypertension, vomiting, alopecia, and dysgeusia. These events are included in the core safety information for pazopanib. The estimated cumulative post-marketing exposure for pazopanib (from 01 October 2009 to 30 September 2010), based on a total daily dose of 800 mg, is 621 patient years.[47]

A total of 247 serious adverse events that had a fatal outcome were reported in 215 subjects as of 09 September 2010. This estimate includes SAEs from GSK sponsored and supported studies, and both placebo, combination therapy, and comparator data. The most frequently reported fatal SAE overall was disease progression, which reporting investigators considered unrelated to study medication in every case. Of the 247 fatal SAEs, 55 subjects had a total of 72 fatal SAEs that the reporting investigator assessed as related to study medication. Amongst these 55 subjects, 22 received pazopanib monotherapy, 25 received pazopanib in combination with other chemotherapy, and 8 received a comparator. The following fatal SAEs assessed as related to study medication by the Investigator were reported in more than 1 subject: pulmonary haemorrhage (4), sudden death (4), dyspnoea (3), pneumonia (3), upper gastrointestinal haemorrhage (3), anaemia (2), bronchopneumonia (2), cardiac arrest (2), hepatic failure (2), hepatic function abnormal (2), peritonitis (2), sepsis (2), thrombocytopenia (2), and vomiting (2).[47] Many of these events are expected with pazopanib therapy, including pulmonary haemorrhage, gastrointestinal haemorrhage, hepatic failure and abnormal hepatic function, thrombocytopenia, and vomiting. Others are not unexpected given combination chemotherapy or background disease in the cancer populations under study.[47]

As of 09 September 2010, there have been 22 deaths reported from the marketed use of pazopanib. In the majority of cases (most reported by a consumer and not medically verified by a healthcare professional), there was insufficient information available to make a clinical assessment regarding the cause of death. For the remainder, the death was likely associated with advanced clinical condition and disease progression. For the majority of reports, no autopsy information was provided.[47] There have been no reports of pregnancy exposures from pazopanib oncology clinical trials or marketed use as of 09 September 2010.[47]

Pazopanib Pharmacokinetics and Pharmacology

The oral bioavailability of pazopanib reflects absorption that is limited by solubility above doses of 800 mg once daily; therefore, increases in doses above 800 mg, up to the highest dose evaluated (2000 mg), in the fasted state will not result in increased systemic exposure. Geometric mean pazopanib t½ values ranged from 18.1 to 52.3 hours. The mean t½ was 30.9 hours in the 800 mg once daily dose group, the monotherapy dose selected for administration in Phase II and Phase III clinical trials. The tmax was 3 to 4 hours. The oral bioavailability of pazopanib may also be affected by its substrate

affinity for gut efflux transporters. Oral absorption is significantly enhanced when pazopanib is dosed with food; therefore, it is recommended to administer pazopanib on an empty stomach, at least 1 hour before or 2 hours after a meal. The absolute bioavailability of pazopanib (median 21%) suggests that the majority (67%) of the oral dose recovered unchanged in feces represents unabsorbed drug.[47]

Pazopanib is not extensively metabolized and first-pass metabolism is minor, consistent with its low plasma clearance and small volume of distribution. Pazopanib is metabolized primarily by CYP3A4 and systemic exposure to pazopanib is altered by inhibitors and inducers of this enzyme. The concomitant use of strong CYP3A4 inhibitors should be avoided. If co-administration of a strong CYP3A4 inhibitor is warranted, a dose reduction to 400 mg of pazopanib is recommended. Grapefruit, which has also been shown to inhibit CYP3A4, may also increase plasma concentrations of pazopanib and should be avoided. CYP3A4 inducers such as rifampin may decrease plasma pazopanib concentrations. Selection of an alternate concomitant medication with no or minimal enzyme induction potential is recommended. The metabolites of pazopanib are produced in low abundance and are therefore unlikely to contribute to its pharmacological activity.[47]

Age, race, and gender had no effect on pazopanib pharmacokinetics (PK). Renal impairment is not expected to influence pazopanib exposure, and dose adjustment is not necessary. Based on baseline hepatic function, 800 mg pazopanib is recommended for patients with mild impairment, and 200 mg for patients with moderate impairment.[47] There were no clinically meaningful changes in QTc interval following pazopanib in a dedicated QT Holter study.[47]

Pharmacodynamic data indicate that pazopanib, at a monotherapy dose of 800 mg once daily, results in effects consistent with inhibition of the VEGF receptors and angiogenic factors it was designed to target (i.e., increases in VEGF and decreases in soluble VEGFR2). Concentration-effect relationships were observed between trough plasma pazopanib concentrations and the development of hypertension in the First Time in Human study as well as in the percent change from baseline in Human study, as well as in the percent change from baseline in soluble VEGFR2 nadir in the Phase II study in renal cell carcinoma. The trough plasma pazopanib concentrations associated with one-half the maximal effect (EC50) in both concentration-effect relationships were similar (15.3 µg/mL for hypertension and 21.3 µg/mL for sVEGFR2), which demonstrates that there is a consistent inhibition of VEGF receptor(s) in subjects with cancer when plasma pazopanib concentrations are maintained above 15 µg/mL.[47]

Pazopanib is currently under development by GSK for the treatment of a variety of human cancers in adults. Currently, pazopanib is administered orally at 800 mg daily in Phase II and Phase III monotherapy studies. In combination therapy, doses ranged from 200 to 800 mg daily in a Phase I combination dose-ranging study in which pazopanib was administered in combination with the GSK compound lapatinib (GW572016). Pazopanib 200 to 800 mg daily is being investigated in combination with multiple cytotoxic chemotherapy regimens.[47]

Radioiodine ^{131}I

Radioiodine ^{131}I (RAI) has been used in the management of well-differentiated papillary and follicular thyroid cancer subtypes for decades.[52] It is taken up and concentrated in thyroid cells through a sodium-iodide membrane transporter and likely retained as organified iodine through the action of thyroid peroxidase. As there is lower expression of this transporter in thyroid cancer cells, there is less iodine retention in thyroid cancer tissue than in normal thyroid tissue.[12,53,54] Radioiodine is often used adjuvantly post surgery or used to treat residual, recurrent or metastatic thyroid cancer. Patients with thyroid cancer that are able to concentrate ^{131}I generally have a better prognosis than those patients that lose the ability to concentrate or take up radioiodine.[53] In fact, the 5 year survival in patients with WDTC pulmonary metastases is much higher (60%) in patients who are sensitive and take up ^{131}I than those who are refractory (30%).[55,56] Skeletal metastases are less sensitive to ^{131}I, possibly due to poor concentration of RAI. Some studies have suggested that metastatic lesions positive on FDG PET scans

are refractory to RAI therapy even if iodine-avid.[13] Generally, the cumulative doses of RAI range from 100 to 600 mCi in patients with metastatic disease.[52] Patients with lymph node and mediastinal disease are generally treated with 150 mCi, patients with pulmonary metastases are generally treated with 150 to 200 mCi and patients with skeletal disease and other distant metastatic disease are usually treated with 200 mCi of RAI, or higher if guided by radiation dosimetry.[52.] However, RAI is not without side effects. Acutely, nausea, vomiting and dehydration are common but preventable and treatable with antinauseants and hydration; whereas, radiation thyroiditis, painless neck edema, sialadenitis and tumor hemorrhage or edema occur less commonly in 10-20% of patients, and are seen predominantly at higher doses.[29,30] Chronically, there is a very small risk of future secondary malignancies such as bladder, salivary, gastrointestinal tract cancers and possibly breast cancer and acute myeloid leukemia.[29,30] Moreover, gonadal dysfunction, particularly ovarian failure in woman can occur, as well as obstruction of the nasolacrimal duct causing excessive tearing. Many of the chronic risks increase with higher cumulative doses.[29,30] Furthermore, the risks of exposure to family members should be minimized by avoidance of close contact with family members for two days post treatment.[52]

3.0 STUDY POPULATION AND SELECTION OF SUBJECTS

3.1 Selection of Subjects: Inclusion Criteria

Deviations from inclusion criteria are not allowed because deviations can potentially jeopardize the scientific integrity of the study, regulatory acceptability, or subject safety. Therefore, adherence to the criteria as specified in the protocol is essential.

1) Subjects must provide written informed consent prior to performance of study-specific procedures or assessments, and must be willing to comply with treatment and follow-up. Procedures conducted as part of the subject’s routine clinical management (e.g., blood count, imaging study) and obtained prior to signing of informed consent may be utilized for screening or baseline purposes provided these procedures are conducted as specified in the protocol.
2) Age ≥ 18 years or legal age of consent if greater than 18 years
3) Histologically confirmed diagnosis of WDTC, including papillary and follicular subtypes, and documented recurrent and/or metastatic disease. Patients must have unresectable disease: patients must not be amenable to surgery but prior thyroidectomy is allowed.
4) Patient must have demonstrated evidence of disease progression by RECIST criteria (Appendix D) using site assessment of CT/MRI scans within 12 months (+1 month to allow for variances in patient scanning intervals) prior to study entry or by >50% increase in suppressed thyroglobulin levels during this time period.
5) Patients with WDTC must be relatively ^{131}I refractory/resistant as defined by at least one of the following:
   a) One or more measurable lesions with low or absent ^{131}I uptake on the most recent pre-study radioiodine scans, based on a visual review of scans or RAI scan reports.
   b) One or more measurable lesions with disease progression by RECIST within 12 months (+ 1 month to allow for variances in patient scanning intervals) of ^{131}I therapy despite ^{131}I uptake on RAI scan, based on site assessment of CT/MRI scans or by >50% increase in suppressed thyroglobulin levels during this time period.
   c) Evidence of at least one site of known disease with preserved ^{131}I uptake above background levels on a diagnostic post-therapy ^{131}I scan prior to study entry.
   d) Patients with WDTC must be receiving thyroxine suppression therapy and TSH should not be elevated (TSH should be ≤ 5.50 mcu/mL).

6) Eastern Cooperative Oncology Group (ECOG) performance status of 0-2. (Appendix C)

7) Adequate organ system function as defined in Table 1 below.

Table 1 Definitions for Adequate Organ Function

System	Laboratory Values
**Hematologic**	
Absolute neutrophil count (ANC)	≥1.5 X 10^9/L
Hemoglobin	≥9 g/dL (5.6 mmol/L)
Platelets	≥90 X 10^9/L
International normalized ratio (INR)[b]	≤1.2 X ULN
Activated partial thromboplastin time (aPTT)	≤1.2 X ULN
**Hepatic**	
Total bilirubin	≤1.5 X ULN
Alanine amino transferase (ALT) and Aspartate aminotransferase (AST)[c]	≤2.5 X ULN, and <5X ULN in the presence of liver metastases
**Renal**	
Serum creatinine	≤2.0 mg/dL
Or, if serum creatinine >2.0 mg/dL, calculate creatinine clearance (Cl_{CR}) as per Appendix G	≥30 mL/min
Urine Protein to Creatinine Ratio (UPC; Appendix F)[d]	<1
Or, 24-hour urine protein	<1g

a. Subjects may not have had a transfusion within 7 days of screening assessment.
b. Subjects receiving anticoagulant therapy are eligible if their INR is stable and within the recommended range for the desired level of anticoagulation.
c. Concomitant elevations in bilirubin and AST/ALT above 1.5 x ULN (upper limit of normal) are not permitted.
If UPC ≥1, then a 24-hour urine protein must be assessed. Subjects must have a 24-hour urine protein value <1 g to be eligible. Use of urine dipstick for renal function assessment is not acceptable.

8) A female is eligible to enter and participate in this study if she is of:
   a) Non-childbearing potential (i.e., physiologically incapable of becoming pregnant), including any female who has had:
      - A hysterectomy
      - A bilateral oophorectomy (ovariectomy)
      - A bilateral tubal ligation
      - Is post-menopausal (Subjects not using hormone replacement therapy (HRT) must have experienced total cessation of menses for ≥ 1 year and be greater than 45 years in age, OR, in questionable cases, have a follicle stimulating hormone (FSH) value >40 mIU/mL and an estradiol value < 40pg/mL (<140 pmol/L). Subjects using HRT must have experienced total cessation of menses for >= 1 year and be greater than 45 years of age OR have had documented evidence of menopause based on FSH and estradiol concentrations prior to initiation of HRT)

b) Childbearing potential, including any female who has had a negative serum pregnancy test within 2 weeks prior to the first dose of study treatment, preferably as close to the first dose as possible, and agrees to use adequate contraception for at least 2 weeks following the last dose of the investigational product.

GSK acceptable contraceptive methods, when used consistently and in accordance with both the product label and the instructions of the physician, are as follow:

- Complete abstinence from sexual intercourse for 14 days before exposure to investigational product, through the dosing period, and for at least 21 days after the last dose of investigational product
- Oral contraceptive, either combined or progestogen alone
- Injectable progestogen
- Implants of levonorgestrel
- Estrogenic vaginal ring
- Percutaneous contraceptive patches
- Intrauterine device (IUD) or intrauterine system (IUS) with a documented failure rate of less than 1% per year
- Male partner sterilization (vasectomy with documentation of azoospermia) prior to the female subject's entry into the study, and this male is the sole partner for that subject
- Double barrier method: condom and an occlusive cap (diaphragm or cervical/vault caps) with a vaginal spermicidal agent (foam/gel/film/cream/suppository)

9) Female subjects who are lactating should discontinue nursing prior to the first dose of study drug and should refrain from nursing throughout the treatment period and for 14 days following the last dose of study drug.

3.2 Selection of Subjects: Exclusion Criteria

Deviations from exclusion criteria are not allowed because they can potentially jeopardize the scientific integrity of the study, regulatory acceptability or subject safety. Therefore, adherence to the criteria as specified in the protocol is essential.

1) Patients with medullary thyroid cancer, thyroid lymphoma or anaplastic thyroid cancer are excluded.
2) Resolution of all acute toxic effects of prior systemic therapy (including iodine therapy or systemic therapy), radiotherapy or surgical procedure to NCI CTCAE version 4.0 (Appendix E) grade ≤ 1. ..
3) Patients with cumulative ^{131}I exposure in excess of 1000 mCi.
4) Second primary malignancy that is of clinical significance, clinical detectable and/or progressing at the time of consideration for study enrollment.
5) History or clinical evidence of central nervous system (CNS) metastases or leptomeningeal carcinomatosis, except for individuals who have previously-treated CNS metastases, are asymptomatic, and have had no requirement for steroids for 28 days prior to first dose of study drug. Screening with CNS imaging studies (computed tomography [CT] or magnetic resonance imaging [MRI]) is required only if clinically indicated or if the subject has a history of CNS metastases.
6) Clinically significant gastrointestinal abnormalities that may increase the risk for gastrointestinal bleeding including, but not limited to:

- Active peptic ulcer disease
- Known intraluminal metastatic lesion/s with risk of bleeding
- Inflammatory bowel disease (e.g. ulcerative colitis, Crohn's disease), or other gastrointestinal conditions with increased risk of perforation
- History of abdominal fistula, gastrointestinal perforation, or intra-abdominal abscess within 28 days prior to beginning study treatment

7) Clinically significant gastrointestinal abnormalities that may affect absorption of investigational product including, but not limited to:

- Malabsorption syndrome
- Major resection of the stomach or small bowel.

8) Presence of uncontrolled infection.

9) Corrected QT interval (QTc) > 480 msecs using Bazett's formula

10) History of any one or more of the following cardiovascular conditions within the past 6 months:

- Cardiac angioplasty or stenting
- Myocardial infarction
- Unstable angina
- Coronary artery bypass graft surgery
- Symptomatic peripheral vascular disease
- Class III or IV congestive heart failure, as defined by the New York Heart Association (NYHA)

11) Poorly controlled hypertension [defined as systolic blood pressure (SBP) of ≥ 140 mmHg or diastolic blood pressure (DBP) of ≥ 90mmHg].

- Note: Initiation or adjustment of antihypertensive medication(s) is permitted prior to study entry.

12) History of cerebrovascular accident including transient ischemic attack (TIA), pulmonary embolism or untreated deep venous thrombosis (DVT) within the past 6 months. Subjects with recent DVT who have been treated with therapeutic anti-coagulating agents for at least 6 weeks are eligible

13) Prior major surgery or trauma within 28 days prior to first dose of study drug and/or presence of any non-healing wound, fracture, or ulcer (procedures such as catheter placement not considered to be major).

14) Evidence of active bleeding or bleeding diathesis.

15) Known endobronchial lesions and/or lesions infiltrating major pulmonary vessels that increase the risk of pulmonary hemorrhage.

    Note: Lesions infiltrating major pulmonary vessels (contiguous tumor and vessels) are excluded; however, the presence of a tumor that is touching, but not infiltrating (abutting) the vessels is acceptable (CT with contrast is strongly recommended to evaluate such lesions).

16) Recent Hemoptysis in excess of 15 ml of bright red blood in the 8 weeks prior to study entry.

17) Any serious and/or unstable pre-existing medical, psychiatric, or other condition that could interfere with subject's safety, provision of informed consent, or compliance to study procedures.

18) Unable or unwilling to discontinue use of prohibited medications listed in §5.8 for at least 14 days or five half-lives of a drug (whichever is longer) prior to the first dose of study drug and for the duration of the study.

19) Treatment with any of the following anti-cancer therapies:

- radiation therapy, surgery or tumor embolization within 14 days prior to the first dose of pazopanib OR

- chemotherapy, immunotherapy, biologic therapy, investigational therapy or hormonal therapy within 14 days or five half-lives of a drug (whichever is longer) prior to the first dose of pazopanib

4.0 TRIAL DESIGN AND TREATMENT PLAN

4.1 Trial Design

This is a phase I open-label, dose-finding, safety study of pazopanib in combination with escalating doses of radioiodine ^{131}I in patients with advanced or recurrent WDTC who still maintain some uptake on RAI scans. This will be a single center study, un-controlled, non-comparative and non-randomized trial with no stratification.

4.2 Endpoints

ENDPOINTS:

- **The *primary end point*** of this phase I study is to assess the toxicity and the occurrence of dose limiting toxicity (DLT) when pazopanib is given in conjunction with radioiodine to establish the MTD and RP2D in combination.
- ***Secondary endpoints*** include tumor response (RECIST criteria) and TTP. Tumor response and TTP will be compared to responses and duration of TTP after last prior historical RAI treatment.
- ***Tertiary endpoints*** involve assessing increased radioiodine uptake, retention (through comparison of radioiodine scans in prior historical studies) and correlative effects of pazopanib on tumor blood flow and response in WDTC (assessed by dynamic FDG-PET).

4.3 DOSE RATIONALE

4.3.1 Treatment Regimen Pazopanib Monotherapy

Pazopanib 800 mg once daily is the recommended monotherapy dose based on clinical and preclinical results. Once daily doses of 50 mg to 2000 mg pazopanib were investigated in the "First Time in Human", Phase I Study VEG10003. Increases in the pazopanib dose above 800 mg once daily when administered in the fasted state did not result in a consistent increase in systemic exposure at steady-state. Therefore, no further benefit is expected at pazopanib doses above 800 mg once daily.[47]

Pharmacodynamic data indicate that pazopanib, at a monotherapy dose of 800 mg once daily, results in effects consistent with inhibition of the VEGF receptors it was designed to target. Concentration-effect relationships were observed between trough plasma pazopanib concentrations and the development of hypertension in Study VEG10003 and the percent change from baseline in sVEGFR2 nadir in Study VEG102616. The trough plasma pazopanib concentrations associated with one-half the maximal effect (EC_{50}) in both concentration-effect relationships were similar (21.3 μg/mL and 15.3 μg/mL) and demonstrate that there is a consistent inhibition of VEGF receptor(s) in subjects with cancer when plasma pazopanib concentrations are maintained above 15 μg/mL. The plasma pazopanib EC_{50} values for biologic effects observed in the clinical studies are similar to the plasma concentration of 40 μM (17.5 μg/mL) required for optimal inhibition of VEGFR-2 phosphorylation in mice.[47]

Progression Free Survival (PFS) in subjects with renal cell cancer in Study VEG102616 was compared between subjects whose trough plasma pazopanib concentrations (Cmin) at Week 4 were above or below selected threshold values. The deciles of the observed Cmin values were selected as threshold values so that approximately equal numbers of subjects were included in each Cmin interval. Subjects with a Cmin at Week 4 above the threshold values had significantly better PFS, compared to the remaining subjects, when the threshold concentrations were 12.6 μg/mL, 17.4 μg/mL, and 20.6 μg/mL. Use of thresholds higher than 21 μg/mL did not result in a significant improvement in PFS between patients with Cmin values above and below the threshold. Patients with Cmin concentrations above 20.6 μg/mL also had a significantly better response rate and tumor shrinkage than the remaining patients.[47]

Pazopanib C24 at steady-state was greater than 15 μg/mL in 93% of subjects who received 800 mg once daily in Study VEG10003. Individual subjects receiving pazopanib doses below 800 mg once daily can achieve plasma concentrations over 15 μg/mL, albeit at a lower frequency compared with what is observed at 800 mg once daily. Therefore, the pharmacokinetic and pharmacodynamic results across clinical studies demonstrate that pazopanib 800 mg once daily results in plasma concentrations that provide optimal biologic effects associated with VEGFR inhibition in the greatest proportion of subjects.[47]

Additional support for an 800 mg once daily pazopanib dose comes in results from Study VEG105192, a 435-subject Phase III study of pazopanib (800 mg once daily) versus placebo in treatment-naïve and cytokine-pretreated subjects with renal cell carcinoma (RCC). In this study, the median progression-free survival (PFS) in the pazopanib arm was 9.2 months (95% CI, 7.4, 12.9) compared to 4.2 months (95% CI, 2.8, 4.2) in the placebo arm. This finding represented a statistically significant improvement in PFS in response to pazopanib monotherapy (HR 0.46, 95% CI 0.34 to 0.62, p<0.0000001). In addition, the response rate (defined as the percentage of subjects achieving either a confirmed complete or partial response according to RECIST) in the pazopanib arm was 30% versus 3% in the placebo arm, and the median duration of response in pazopanib-treated subjects was 58.7 weeks. Results from Study VEG105192 therefore indicate that an 800 mg once daily dose of pazopanib is highly effective in treating subjects with advanced RCC.[47]

4.3.2 Treatment Regimen Radioiodine ^{131}I

The radioiodine dosing will be based upon estimated radiation exposure to the blood as an indicator of the marrow radiation dose using the maximal dose dosimetry method.[53,54,57] We have been routinely using this method for WDTC patients with distant metastases over 15 years and over 500 patients. For this method, in the absence of concurrent therapy, the maximum tolerated dose with severe marrow toxicity has been show to be 200 rads.[53,54,57] As additive hematologic toxicity may occur in combination with pazopanib, the starting dose of ^{131}I will be at 50 rads to the blood as estimated by dosimetry based upon radioiodine whole-body and blood clearance measures and escalation will proceed up the clinically established single agent dose of 150 rads. The combined effect of pazopanib on hematologic and non-hematologic toxicity, particularly marrow and pulmonary toxicity, on ^{131}I will be assessed in this study.

4.4 Treatment Plan

The clinically efficacious and tolerable phase II dose of pazopanib will be fixed at 800 mg po once daily and initiated for 4 weeks before adding in combined escalating doses of ^{131}I radioiodine in a 3+3 phase I design according to Table 2 below, starting at Dose Level 1. Pazopanib continued to be will be administered once daily for 8 weeks post radioiodine therapy, for a total of 12 weeks of therapy. During the 4 week run-in period, if grade ≥ 3 pazopanib-related persistent toxicities occur despite supportive

measures, patients may continue on study at the Investigator's discretion with a reduced dose of to 600 mg po once daily concurrent with escalating doses of ^{131}I, which will be continued post RAI. The radioiodine dosing will be based upon estimated radiation exposure to the blood as an indicator of the marrow radiation dose using the maximal dose dosimetry method.[53,54,57] We have been routinely using this method for WDTC patients with distant metastases over 15 years and over 500 patients. For this method, in the absence of concurrent therapy, the maximum tolerated dose with severe marrow toxicity has been show to be 200 rads.[53,54,57] As additive hematologic toxicity may occur in combination with pazopanib, the starting dose of ^{131}I will be at 50 rads and escalation will proceed up the clinically established single agent dose of 150 rads, slightly below the accepted marrow toxicity limit of up to 200 rads to the blood for patients not receiving concurrent systemic therapy. The combined effect of pazopanib on hematologic and non-hematologic toxicity, particularly marrow and pulmonary toxicity, on ^{131}I will be assessed in this study.

4.4.1 Drug Regimen:

Pazopanib at a fixed dose will be administered for 4 weeks before adding in combined escalating doses of ^{131}I radioiodine in a 3+3 phase I design according to Table 2 below, starting at Dose Level 1. Pazopanib will continue for 8 weeks post radioiodine therapy, for a total of 14 weeks of therapy. The radioiodine dosing will be based upon estimated radiation exposure to the blood as an indicator of the marrow radiation dose using the maximal dose dosimetry method.[53,54,57] Severe marrow toxicity has been observed at 200 rads using this method.[53,54,57] As additive hematologic toxicity may occur in combination with pazopanib, the starting dose of ^{131}I will be at 50 rads and escalation will proceed up our center's typical clinical dose of 150 rads for ^{131}I alone.

Table 2.0 Dose levels for pazopanib in combination with ^{131}I

Dose level -2	Pazopanib 600 mg po once daily continuous dosing<br>Radioiodine ^{131}I 25 rad therapy
Dose level -1	Pazopanib 600 mg po once daily continuous dosing<br>Radioiodine ^{131}I 50 rad therapy
Dose level 1<br>(Starting Dose)	Pazopanib 800 mg po once daily continuous dosing<br>Radioiodine ^{131}I 50 rad dose of therapy
Dose level 2	Pazopanib 800 mg po once daily continuous dosing<br>Radioiodine ^{131}I 100 rad dose of therapy
Dose level 3	Pazopanib 800 mg po once daily continuous dosing<br>Radioiodine ^{131}I 125 rad dose of therapy
Dose level 4	Pazopanib 800 mg po once daily continuous dosing<br>Radioiodine ^{131}I 150 rad dose of therapy

4.4.2 Administration of Pazopanib

Pazopanib will be provided by GSK.

Pazopanib should be taken orally without food at least one hour before or two hours after a meal. The tablets should be swallowed whole and must not be crushed or broken. The time of day the tablets are taken should be relatively constant. If a dose is missed, the subject should take the dose as soon as possible, but only if there are 12 or more hours remaining before the next dose is due. If the next dose

is due in less than 12 hours, the subject should skip the missed dose and take the next dose as scheduled.

If vomiting occurs after taking pazopanib, the subject should not take a replacement dose on that day. The subject should resume taking pazopanib at the next scheduled dose on the following day. If vomiting persists, the subject should be instructed to notify the Investigator.

Handling and Storage of Study Treatment

Pazopanib should be stored at room temperature up to 25°C. When stored at these temperatures and in unopened bottles, pazopanib tablets will remain stable until the expiration date indicated on the bottle label.

4.4.3 Administration of Radioiodine ^{131}I

The radioiodine dosing will be based upon estimated radiation exposure to the blood as an indicator of the marrow radiation dose using the maximal dose dosimetry method.[53,54,57] This is a standard RAI administration method used throughout North America.

4.5 Treatment Schedule

Study treatment will proceed as per the schema outlined in Appendix A. A lead-in period of 4 weeks starting with pazopanib at 800 mg po once daily alone will be conducted to assess for severe toxicities prior to initiating RAI therapy. If severe (≥ Grade 3) toxicities which cannot be controlled by medical management and supportive care measures, or major uncorrectable laboratory abnormalities - occur with pazopanib alone, patients may need to stop drug and once toxicities resolve to ≤ Grade 1, patients may continue pazopanib therapy at a reduced dose of 600 mg po once daily prior to receiving RAI therapy. Patients who require pazopanib treatment to be held for greater than 14 days prior to RAI therapy, will be taken off study. Patients should have received at least 14 consecutive days of pazopanib prior to starting RAI. In the event of Grade 2 toxicities which are deemed intolerable by the patient at the 800 mg po daily dose, patients may also continue pazopanib at a reduced dose of 600 mg po once daily prior to receiving RAI therapy at the discretion of the Investigator. Patients who cannot tolerate the 600 mg po once daily dosing prior to receiving RAI therapy, will be considered unevaluable and be replaced. Patients who do not receive any RAI therapy will be unevaluable and replaced. Patients whose pazopanib dose has been reduced, should continue at the reduced dose for the remainder of the study and no intra-patient re-dose escalation will be allowed.

Pazopanib therapy will be administered continuously throughout the RAI therapy and for a total of 8 weeks post RAI treatment for a total of approximately 14 weeks of therapy. Pazopanib toxicities will be observed throughout the treatment period and the 8 weeks of pazopanib therapy post RAI treatment will serve as the dose-limiting toxicity (DLT) assessment period.

Dose modifications, dose delays and other toxicities will be assessed throughout all courses of treatment when determining the optimal dose for phase II studies (RP2D).

<u>Pre-Study Preparation</u>:

Relevant baseline CT/MRI imaging will be performed within 4 weeks of study initiation. Baseline dynamic FDG-PET, suppressed thyroglobulin levels and laboratory assessments and will be performed within 1 week prior to study initiation.

<u>Week 1 Initiate pazopanib:</u>

Pazopanib will be administered starting on Day 1, as per the dose levels above, as a continuous once daily oral dose. Continuous dosing of pazopanib will continue through the preparation and administration of radioactive iodine until 8 weeks post radioactive iodine - for a total of approximately 14 weeks of therapy.

Week 3 to 4 Initiation of low iodine diet:

A low iodine diet should be initiated at least two weeks prior to starting diagnostic scan for RAI therapy.

Guidelines for a low iodine diet:

Consume less than 50 mcg iodine per day.
Avoid foods high in iodine (over 20 mcg per serving)
Limit the quantity of food moderate in iodine (5 to 20 mcg per serving)
Eat any foods low in iodine (up to 5 mcg per serving)

Weeks 4 to 5:

After completing 3 -3.5 weeks of continuous pazopanib therapy, repeat dynamic FDG-PET imaging will be performed to assess the PET uptake, tumor perfusion (from FDG delivery rates) and response in WDTC.

Weeks 5 to 7: RAI therapy with continued daily dosing of pazopanib

A ***diagnostic scan*** to determine uptake of radioiodine by thyroid tissue will be performed with whole body imaging and dosimetric calculations using a low dose of 2 to 5 mCi ^{131}I. Dosimetric calculations are performed by measuring whole body (WB) and blood ^{131}I clearance.[53,54,57] Localization of uptake is determined with WB scans taken pre- and post-therapy and compared to each patient's prior historical control obtained in the absence of pazopanib. Dosemetric scans will be compared to the baseline historical evaluations to determine the effects on increased iodine uptake, radiosensitization and possible response. In addition, WB and blood clearance ^{131}I will be compared to look for effects of pazopanib on iodine kinetics.

RAI preparation: For RAI therapy, our center will only use the rhTSH-stimulated (Thyrogen-stimulated) preparation methods in this study for maximal dose dosimetry treatments to avoid possible issues of tolerability inherent with total hormone withdrawal while on pazopanib. Preparation for the RAI therapy for this study will use recombinant rhTSH-stimulated (Thyrogen-stimulated) preparation methods and compare this to the patient's most recent prior iodine therapy to allow for determination of radioiodine uptake changes in the presence of pazopanib.

The recombinant rhTSH method requires the administration of rhTSH several days prior to RAI therapy at a dose of 0.9 mg IM once daily for two days prior to the diagnostic radioiodine dose (typically, this should fall on a Saturday or Sunday). The diagnostic dose is then given, followed by 5 days (Monday to Friday) of dosimetry/scanning. Generally, the same timing is used for the diagnostic dose given for scanning/dosimetry measurement and again for the therapeutic dose. Specifically, the next week, patients will receive rhTSH 0.9 mg IM once daily for two days prior to the treatment therapeutic dose (typically a Monday or Tuesday), then patients will be given a therapeutic dose and this will be given an inpatient as patients are admitted for isolation to avoid radiation exposure. Patients remain on thyroxine suppression throughout the process and no thyroid hormone withdrawal is needed. We expect most patients to be admitted for radiation isolation after therapeutic radioiodine. Patients will not be hospitalized for radiation isolation if they are treated with less than 200 mCi and are able to comply with regulations that limit the exposure to others including the use of their own bathroom for 3-4 days.

Patients unable to comply with these regulations, including patients who live with pregnant women or children that cannot avoid being close to the patient will be admitted. Hospital admission occurs from dosing until the exposure rate is safe for release (less than 7mrem/hr at one meter), typically 1-2 days, almost never > 3 days.

Radioiodine Dose: The administered radio-iodine activity (mCi) dose will be determined from the estimated blood radiation dose from clearance measurements, in escalating doses from 50 to 150 rads (see Table 2.) will be administered in the presence of chronic pazopanib therapy. In patients with diffuse lung disease, calculations will also assure that patients are under the anticipated dose for pulmonary toxicity, 80 mCi retained at 2 days,[54] (by the same proportion as the marrow dose compared to 200 rads), for any given dose escalation level. If needed, the dose will be reduced to avoid a potentially lung-toxic dose.

Weeks 7 to 8: Post-treatment scanning:

Tumor uptake of the treatment dose of ^{131}-I will be confirmed with a whole body scan appropriately 1 week (7 – 10 days) post treatment dose of RAI therapy. Post-treatment scans will be compared to pretreatment scans and also historical post-treatment scans in the absence of pazopanib therapy.

Weeks 7 to 15:

The acute toxicities and hematologic toxicities of RAI are prominent for 6 to 8 weeks post RAI administration. Pazopanib will continue as a daily therapy for a total of an additional 8 weeks post RAI before being discontinued. Regular bi-monthly or monthly assessment of blood counts will be performed during this period. Toxicity assessments and the DLT evaluation period will occur up to 8 weeks post RAI therapy.

Week 15 + :

Pazopanib therapy is discontinued and post-treatment follow-up is initiated. Tumor imaging and suppressed thyroglobulin levels will be performed within a week of discontinuing pazopanib therapy to establish a post-treatment follow-up baseline. Radiologic and suppressed thyroglobulin monitoring will occur regularly at 3 to 6 months intervals or per institutional standard of care intervals or as deemed necessary and relevant by the Investigator. Post therapy tumor response and TTP will be determined with respect to censored post pazopanib therapy baseline imaging and thyroglobulin levels. Progression will be determined by clinical radiologic progression by RECIST criteria compared to radiologic imaging post completion of pazopanib and/or increase in suppressed serum thyroglobulin levels >50% with comparison to post-pazopanib levels.

Figure 1: Treatment Schema

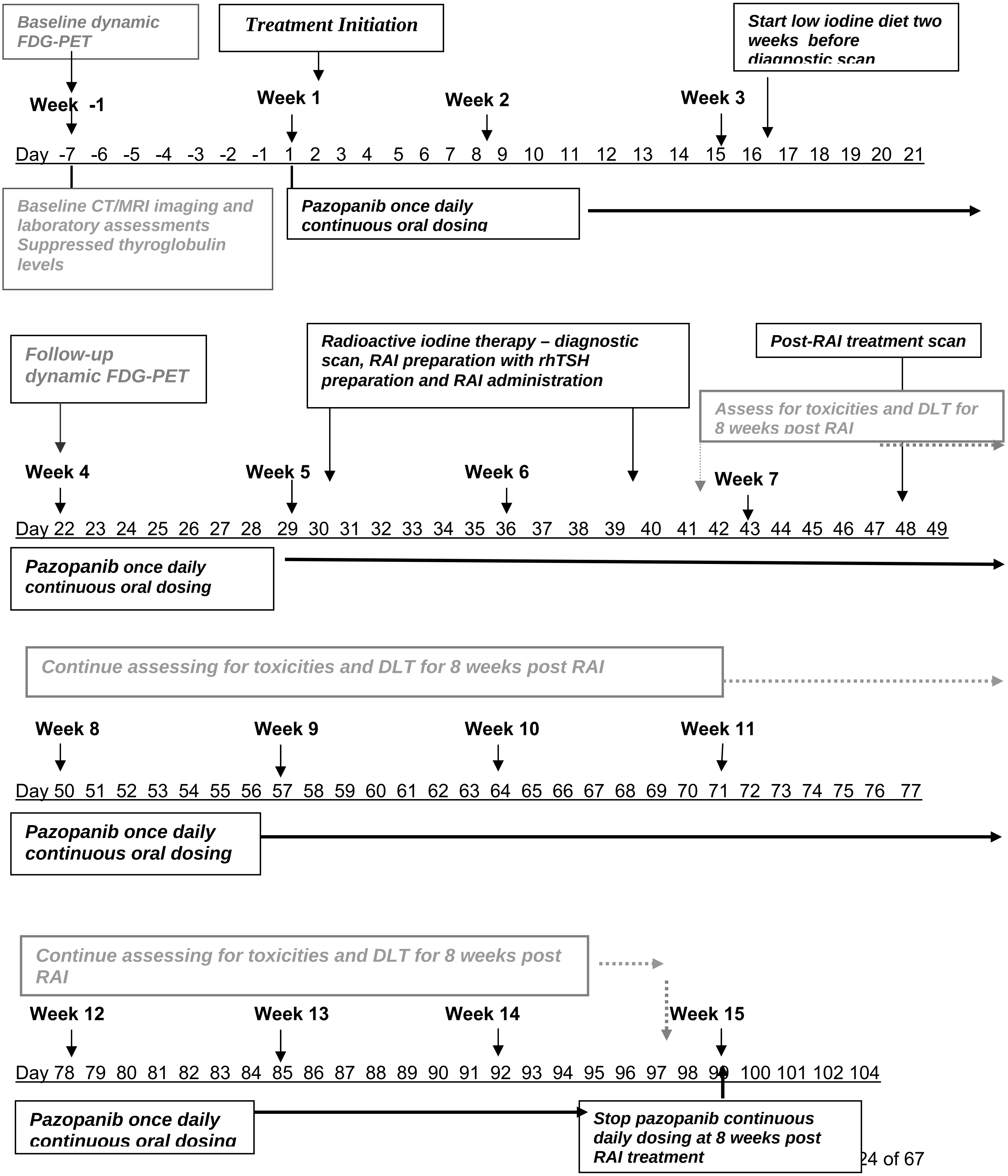

5.0 STUDY CONDUCT AND TRIAL PROCEDURES

Prior to undergoing any study-specific procedure, patients must read and sign the current Institutional Review Board (IRB)-approved informed consent form. Procedures conducted as part of the subject's routine clinical management (e.g., blood count, imaging study) and obtained prior to signing of informed consent may be utilized for screening or baseline purposes provided these procedures are conducted as specified in the protocol. All on-study procedures are permitted within the window frame indicated in Schedule of Protocol Activities (Appendix A).

5.1 Screening

The following screening procedures should be performed within 7 days prior to initiation of treatment on-study unless otherwise specified:

- Patient signature on current IRB-approved informed consent form. May be obtained up to 30 days prior to treatment.
- Demographics specifically including date of birth, race and gender.
- Medical history including and specifying:
  - Tumor-specific history including: date of diagnosis, primary tumor type with histology/cytology determination, current stage of cancer, prior treatment(s) for WDTC (systemic, surgical and/or radiotherapy), ongoing toxicity related to prior treatment(s); and history of other malignancies.
  - Current medical conditions.
- ECOG performance status.
- Height (only recorded at baseline) body weight, and vital signs (temperature, blood pressure, heart rate, respiratory rate).
  - Note: If a subject presents with poorly controlled hypertension, defined as SBP ≥140 mmHg or DBP ≥ 90 mmHg, antihypertensive medication(s) should be initiated or adjusted with a goal to control the blood pressure to <140/90 mmHg.
- Physical examination, including examination of major body systems.
- Hematology and Chemistry as described in Appendix B.
- Baseline thyroglobulin levels, TSH, free T4 and free T3 levels.
- Urine Protein Creatinine ratio (UPC).
- Serum pregnancy test for women of childbearing potential (if applicable). May be performed up to 14 days prior to treatment.
- 12-lead ECG with QTc measurement.
- Baseline Tumor imaging, including dynamic FDG PET scan, CT, or MRI or PET/CT of the chest, and other known sites of disease.
  - May be done up to 28 days prior to treatment.
  - Note that the same imaging modality must be used for each patient throughout the study.

- Brain CT or MRI scan for known (treated) or suspected brain metastases. May be done up to 28 days prior to treatment. Imaging of the brain is not needed in the absence of clinical symptoms or clinical suspicion of brain metastases or CNS involvement.
- Bone scan for patients with known or suspected bone metastases. May be done up to 28 days prior to treatment. Bone scan not needed in the absence of clinical symptoms or lack of clinical suspicion for bone metastases.
- The results of all screening assessments should be reviewed by the Investigator to ensure the patient meets all eligibility requirements as outlined in §3.0. Thereafter, qualified subjects can begin the initial pazopanib treatment.

5.2 Prior to Initiating Pazopanib Therapy on Day 1

- Review of the inclusion/exclusion criteria.
- Physical examination: to identify any changes in the subject's mental and medical conditions since baseline assessment that would make him/her ineligible for the study.
- Blood pressure measurement:
    - It is particularly important to check the blood pressure of any subject for whom anti-hypertensive medication has been initiated and/or dosing has been adjusted during the Baseline Period. At least 24 hours must have elapsed between anti-hypertensive medication initiation or adjustment and BP measurement. If, after treatment with anti-hypertensive medication, a subject's blood pressure is not <140/90mmHg, then further modifications of these medication(s) may be made while the subject is still in the Baseline Period. After further anti-hypertensive treatment, the subject's blood pressure must then be rechecked to determine eligibility for the trial.
- ECOG PS. Subjects having deterioration of ECOG PS to ≥2 will be excluded from the study.
- Review results of all the other baseline assessments to determine the subject's eligibility for the study. All laboratory results must be within the values outlined in the Inclusion Criteria (Section 3.1), otherwise the subject is not eligible to participate in the study.

5.3 Pazopanib Monotherapy Treatment Weeks 1 to 4

Please refer to Appendix A for a comprehensive list of required assessments and procedures.

- Pazopanib will be administered as a standard dose at 800 mg po once daily. Assessment of toxicities due to pazopanib alone will be determined and recorded. If dose reductions are necessary due to toxicity as previously specified, patients may decrease down to 600 mg po once daily continuous dosing.
- Weekly laboratory work may be performed as per local clinical practice. Urine protein creatinine ratio, hematology and chemistry as per Appendix B are suggested.
- Dynamic correlative PET CT scans should be performed by week 4 prior to initiation of RAI

5.4 Reassessment Prior to Initiation of RAI Week 4 to 5

- Low iodine diet must be started 2 weeks prior to initiating diagnostic scans for RAI therapy.
- Reassessment of all toxicities, and all drug-related adverse events.
- ECOG performance status, body weight, and vital signs.
- 12-lead ECG with QTc measurement.
- Physical examination including major body systems.
- Urine Protein Creatinine ratio (UPC).
- Hematology and Blood Chemistry (see Appendix B) if not performed within the previous 7 days.
- Following 4 weeks of pazopanib therapy and prior to initiation of RAI in combination with pazopanib, the following criteria must be met:
  - Patients must have received at least 14 days or more of pazopanib therapy.
  - Patients should not have any current or ongoing ≥ Grade 3 non-hematologic toxicities due to pazopanib alone.
  - o The subject must demonstrate adequate bone marrow, renal, and hepatic function as evidenced by the following:

    o Absolute neutrophil count (ANC) ≥ 1,500 cells/μL

    o Platelet count ≥ 80,000 cells/μL

    o Hemoglobin ≥ 8.0 g/dL

    o Creatinine ≤ 2.0 mg/dL

    o Total bilirubin ≤ 2.0 x ULN

    o SGOT(AST), SGPT (ALT) ≤ 2.5 x ULN

    (for patients with liver metastases, AST, ALT <5x ULN is acceptable)

- Patients who cannot meet the minimal criteria above prior to starting combined RAI therapy, will be replaced.

5.5 Combined Therapy with Pazopanib and RAI Weeks 5 to 7

Refer to Appendix A for a comprehensive list of required assessments and procedures.

Refer to §4.4 for details on cohort enrollment, assignment of RAI dose level, and assessment of dose-limiting toxicities.

- Assessment of adverse events and tumor-related signs and symptoms at each study visit.
- Hematology and blood Chemistry (see Appendix B) according to the schedule in Appendix A.

Weeks 5 to 7: RAI therapy with continued daily dosing of pazopanib

- Assessment of adverse events and tumor-related signs and symptoms at each study visit.

- Hematology and blood Chemistry (see Appendix B) according to the schedule in Appendix A.

A ***diagnostic scan*** to determine uptake of radioiodine by thyroid tissue will be performed with whole body imaging and dosimetric calculations using a low dose of 2 to 5 mCi ^{131}I. Dosimetric calculations are performed by measuring whole body (WB) and blood ^{131}I clearance.[53,54,57] Localization of uptake is determined with WB scans taken pre- and post-therapy and compared to each patient's prior historical control obtained in the absence of pazopanib. Dosemetric scans will be compared to the baseline historical evaluations to determine the effects on increased iodine uptake, radiosensitization and possible response. In addition, WB and blood clearance ^{131}I will be compared to look for effects of pazopanib on iodine kinetics.

RAI preparation: For RAI therapy, our center will only use the rhTSH-stimulated (Thyrogen-stimulated) preparation methods in this study for maximal dose dosimetry treatments to avoid possible issues of tolerability inherent with total hormone withdrawal while on pazopanib. Preparation for the RAI therapy for this study will use recombinant rhTSH-stimulated (Thyrogen-stimulated) preparation methods and compare this to the patient's most recent prior iodine therapy to allow for determination of radioiodine uptake changes in the presence of pazopanib. Patients undergo a two-week low-iodine diet as per clinical routine.

The recombinant rhTSH method requires the administration of rhTSH on the two days prior to RAI therapy at a dose of 0.9 mg IM. Patients remain on thyroxine suppression throughout the process and no thyroid hormone withdrawal is needed.

Radioiodine Dose: The administered radio-iodine activity (mCi) dose will be determined from the estimated blood radiation dose from clearance measurements, in escalating doses from 50 to 150 rads (see Table 2.) will be administered in the presence of chronic pazopanib therapy. In patients with diffuse lung disease, calculations will also assure that patients are under the anticipated dose for pulmonary toxicity, 80 mCi retained at 2 days,[54] (by the same proportion as the marrow dose compared to 200 rads), for any given dose escalation level. If needed, the dose will be reduced to avoid a potentially lung-toxic dose.

Weeks 7 to 8: Post-treatment scanning:

Tumor uptake of the treatment dose of ^{131}-I will be confirmed with a whole body scan appropriately 1 week (7 – 10 days) post therapy. Post-treatment scans will be compared to pretreatment scans and also historical post-treatment scans in the absence of pazopanib therapy.

5.6 Post RAI Therapy with Continued Pazopanib Weeks 7 to 15

- Assessment of adverse events and tumor-related signs and symptoms at each study visit.
- 12-lead ECG with QTc measurement according to the schedule in Appendix A.
- Urine Protein Creatinine ratio (UPC) according to the schedule in Appendix A.
- Hematology and blood Chemistry (see Appendix B) according to the schedule in Appendix A.
    - The acute toxicities and hematologic toxicities of RAI are prominent for 6 to 8 weeks post RAI administration.
- Pazopanib will continue as a daily therapy for a total of an additional 8 weeks post RAI before being discontinued.
- Regular bi-monthly or monthly assessment of blood counts will be performed during this period. Toxicity assessments and the DLT evaluation period will occur up to 8 weeks post RAI therapy.

5.7 Completion of Pazopanib Therapy at 8 weeks post RAI (Week 15)

Pazopanib therapy is discontinued and post-treatment follow-up is initiated.

- Tumor imaging and suppressed thyroglobulin levels will be performed within a week of discontinuing pazopanib therapy to establish a post-treatment follow-up baseline.
- Laboratory assessments of hematology and blood chemistry (including TSH, free T3 and free F4), study visits, clinical disease assessments, assessments of toxicity, physical examination and vitals will occur regularly at 3 to 6 months intervals or per institutional standard of care intervals or as deemed necessary and relevant by the Investigator.
    - o Assessment of adverse events and tumor-related signs and symptoms at each study visit.
    - o ECOG performance status, body weight, and vital signs at each study visit.
    - o Physical examination, including major body systems, at each study visit. A problem-oriented PE can be conducted at the Investigator's discretion at any time.
- Radiologic and suppressed thyroglobulin monitoring will occur regularly at 3 to 6 months intervals or per institutional standard of care intervals or as deemed necessary and relevant by the Investigator.
    - o Tumor imaging, including CT or MRI scans of the chest and other applicable sites of disease (must be the same imaging modality(ies) and anatomical sites as screening assessments.
- Post therapy tumor response and TTP will be determined with respect to censored post pazopanib therapy baseline imaging and thyroglobulin levels.

- Progression will be determined by clinical radiologic progression by RECIST criteria and/or increase in suppressed serum thyroglobulin levels >50% with comparison to post-pazopanib levels.

5.8 Concomitant Medications and Supportive Care

If future changes are made to the list of permitted/prohibited medications, formal documentation will be provided by GSK and stored in the study file. Any such changes will be communicated to the investigative sites in the form of a letter.

Permitted Medications

All subjects will be asked to provide a complete list of prescription and over-the-counter medications that have been taken within the 4 weeks prior to Screening. The Investigator must be informed as soon as possible about any new medication(s) taken from the time of Screening until the completion of the post-treatment follow-up visit.

Subjects should be provided with full supportive care measures as clinically indicated, and in accordance with institutional standards, including transfusion of blood and blood products, and treatment with antibiotics, analgesics, erythropoietin, or bisphosphonates, when appropriate. Anti-emetics (such as prochlorperazine, lorazepam, ondansetron or other 5-HT antagonists) may be administered prophylactically in the event of nausea. Anti-diarrheals, such as loperamide, may be administered as needed in the event of diarrhea. Although acetaminophen at doses of ≤2 g/day is permitted, it should be used with caution in subjects with impaired liver function.

Localized radiotherapy is permitted for palliation of painful lesions at the Principal Investigator's discretion provided it is not within 2 weeks (before or after) of RAI administration

Permitted Medications – Use with Caution

Specific recommendations regarding anticoagulants:
Results from drug-drug interaction studies conducted in subjects with cancer suggest that pazopanib has no effect on the metabolism of S-warfarin. Hemorrhagic events, however, have been reported in clinical studies with pazopanib; therefore, pazopanib should be used with caution in subjects with increased risk of severe bleeding or who are receiving concomitant anticoagulant therapy (e.g., warfarin or its derivatives, low molecular weight heparin, unfractionated heparin). Subjects taking concomitant anticoagulant therapy should be monitored regularly for changes in relevant coagulation parameters as clinically indicated, as well as for any clinical bleeding episodes.

Specific recommendations regarding hypoglycemic therapy including insulin:
Results from drug-drug interaction studies conducted in subjects with cancer suggest that there will be no clinically relevant pharmacokinetic interaction between pazopanib and hypoglycemic agents. Transient decreases in serum glucose (mainly Grade 1 and 2, rarely Grade 3) have been observed in clinical studies with pazopanib. In addition, decreases in blood sugar have been recently reported in subjects treated with another small molecule tyrosine kinase inhibitor, sunitinib.[58] Such changes may require an adjustment in the dose of hypoglycemic and/or insulin therapy. Subjects should be advised to report symptoms of hypoglycemia (e.g., confusion, visual disturbances, palpitations, sweating). Serum glucose should be tested during treatment with pazopanib as outlined in the protocol and as clinically indicated.

The Effects of Pazopanib on Other Drugs
In vitro data indicate that pazopanib is a potential inhibitor for CYP3A4, CYP2C8, CYP2D6, CYP1A2, CYP2C9, CYP2C19, CYP2A6, CYP2B6, and CYP2E1.[47] Pregnane X receptor transient transfection assay suggested some potential for human CYP3A4 induction at high concentrations. Results from drug-drug interaction studies conducted in subjects with cancer suggest that pazopanib is a weak inhibitor of CYP3A4, CYP2C8, and CYP2D6 in vivo, but had no clinically relevant effect on CYP1A2, CYP2C9 or CYP2C19 metabolism. Therefore, concomitant use of pazopanib with certain medications (substrates of CYP3A4, CYP2C8, and CYP2D6) with a narrow therapeutic window should be undertaken with **CAUTION** due to the potential for alterations in the pharmacologic effects of these medications or an increased risk for serious or life threatening adverse events associated with such medications (see below) secondary to the inhibition of specific CYP enzymes by pazopanib. In addition, the potential for drug interaction with such medications, although diminished, may persist after the last dose of pazopanib due to its long half-life (i.e., mean 30.9 hours); therefore, continue to exercise **CAUTION** for at least 7 days and up to 15 days after the last dose of pazopanib when administering these medications. These medications include (but are not limited to):

- Ergot derivatives: dihydroergotamine, ergonovine, ergotamine, methylergonovine (potential increased risk for developing ergot toxicity that includes severe vasospasm leading to peripheral as well as cerebral ischemia).
- Neuroleptics: pimozide (potential increased risk for QT interval prolongation, ventricular arrhythmia, and sudden death)
- Antiarrhythmics: bepridil, flecainide, lidocaine, mexiletine, amiodarone, quinidine, propafenone (potential increased risk for QT interval prolongation and Torsade de Pointes)
- Immune modulators: cyclosporine, tacrolimus, sirolimus (potential increased risk for nephrotoxicity and neurotoxicity)
- Miscellaneous: quetiapine, risperidone, clozapine, atomoxetine.

The Effects of Other Drugs on Pazopanib
Results from in vitro studies suggest that the oxidative metabolism of pazopanib in human liver microsomes is mediated primarily by CYP3A4, with minor contributions from CYP1A2 and CYP2C8. Furthermore, in vitro data suggest that pazopanib is a substrate for p-glycoprotein.[47] Substances that induce or inhibit CYP3A4 may alter the pharmacologic effects of pazopanib and should be used with **CAUTION**.

Medications that inhibit CYP3A4 may result in increased plasma pazopanib concentrations. Co-administration of strong CYP3A4 inhibitors is prohibited (see Section on Prohibited Medications); therefore selection of an alternate concomitant medication with no or minimal potential to inhibit CYP3A4 is recommended.

CYP3A4 inducers may decrease plasma pazopanib concentrations. Selection of an alternate concomitant medication with no or minimal enzyme induction potential is recommended. **Drugs that induce CYP3A4 and may decrease pazopanib plasma concentrations include (but are not limited to)**:

- o <u>Glucocorticoids</u>: cortisone (> 50 mg), hydrocortisone (> 40 mg), prednisone (> 10 mg), methylprednisolone (> 8 mg), dexamethasone (> 1.5 mg)
- o Anticonvulsants: phenytoin, carbamezepine, phenobarbital, oxcarbazepine
- o HIV antivirals: efavirenz, nevirapine
- o Antibiotics: rifampin (rifampicin), rifabutin, rifapentene
- o Miscellaneous: St. John’s Wort, modafinil, pioglitazone

Prohibited Medications

The following medications and interventions are prohibited from the time of study screening until the End of Study visit:

- Subjects should not receive other anti-cancer therapy [cytotoxic, biologic, radiation, or hormonal (other than leuprolide or other GnRH agonists)] while on treatment in this study.
- Any investigational device or drug other than pazopanib
- Medications that inhibit CYP3A4 may result in increased plasma pazopanib concentrations; therefore, co-administration of strong CYP3A4 inhibitors is **PROHIBITED** beginning **14** days prior to the first dose of study drug until discontinuation from the study.

  Strong CYP3A4 inhibitors include (but are not limited to):

  - Antibiotics: clarithromycin, telithromycin, troleandomycin
  - HIV: protease inhibitors (ritonavir, indinavir, saquinavir, nelfinavir, lopinavir)
  - Antifungals: itraconzaole, ketoconazole, voriconazole
  - Antidepressants: nefazodone

5.9 End of Study Treatment / Withdrawal Procedures

Patients will have completed their participation in the study in the case of:

- Disease progression, except as described below
- Unacceptable toxicity
- Need for treatment rest > 14 days; refer to §5.14.1
- Need to reduce dose of pazopanib to lower than 400 mg po daily; refer to §5.14
- Need for anticancer therapy not specified in the protocol
- Patient noncompliance
- Patient lost to follow-up
- Patient choice to withdraw from treatment (follow-up permitted by patient; see below)
- Withdrawal of patient consent (cessation of follow-up; see below)
- Study closure by Sponsor-Investigator

Subjects may withdraw from the trial at any time at their own request, or they may be withdrawn at any time at the discretion of the Investigator or Sponsor for safety, behavioral, or administrative reasons.

If the subject withdraws from the trial and also withdraws consent for disclosure of future information, no further evaluations should be performed and no additional data should be collected. The Sponsor may retain and continue to use any data collected before such withdrawal of consent.

At the end of the study or at withdrawal, the following procedures should be performed if they were not performed during the last week on study:

- ECOG performance status, body weight, and vital signs
- Assessment of adverse events and tumor-related signs and symptoms

- Physical examination including major body systems
- Hematology and Blood Chemistry as described in Appendix B
- ECG, if clinically indicated
- Relevant tumor imaging including bone scans, CT or MRI or PET/CT scans of known sites of disease (utilizing the same imaging modalities and anatomical areas as Screening)

5.10 Follow-up Visit Procedures

Patients should continue to be evaluated for 28 calendar days after the last dose of study treatment. At the post-treatment follow-up visit, the following procedures should be performed:

- Assessment of adverse events and tumor-related signs and symptoms.
- Physical examination, ECOG performance status, body weight, vital signs, laboratory assessments, or other tests necessary to follow unresolved adverse events.

During this period, the outcome of adverse events with a date of onset during the study period should be reevaluated. Adverse events will be followed until they are resolved or until a new anti-cancer treatment is initiated. All serious adverse events, and those non-serious adverse events assessed by the Investigator as possibly related to study drug, should continue to be followed even after patient withdrawal from study. These adverse events should be followed until they resolve or until the Investigator assesses them to be “chronic” or “stable.”

5.11 Cohort Enrollment, Expansion & Replacement of Patients

This study will evaluate pazopanib in combination with RAI in a dose-escalation scheme, whereby cohorts of n=3 patients will be enrolled and administered increasing doses of RAI until the MTD/RP2D is determined.

For each cohort, 3 patients will be enrolled and administered pazopanib and RAI at the assigned dose level. Safety data through week 14 for all 3 patients must be reviewed before enrollment of the next cohort can be considered.

If none of the 3 patients experiences a DLT (see §5.12) after week 14, dose escalation may proceed and 3 new patients may be enrolled in the next cohort (cohort 2) and administered pazopanib plus RAI at the next dose level. Cohort 3 will be enrolled and advanced in this same manner.

If 1 of 3 patients in a cohort experiences a DLT during Cycle 1, the cohort will be expanded and up to 3 additional patients must be treated at that dose level before RAI dose escalation can proceed. If a total of 1 of 6 patients experiences a DLT during Cycle 1, dose escalation may continue. If 2 or more patients in a cohort experience a DLT in Cycle 1, no additional patients will be treated at that dose of RAI, and that dose level will be considered unacceptable. Once the MTD/RP2D (defined as the highest dose level at which 0 of 3 or 1 of 6 patients experiences a DLT in Cycle 1) has been identified, up to 3 additional patients should be enrolled at that dose level of RAI to ensure a total of 6 patients are treated at the RP2D.

Patients will be initiated at Dose Level 1. In the case of ≥ 2 patients with DLT, a de-escalation will occur to enroll patients onto Dose Level -1.

5.12 Dose-Limiting Toxicities (DLT)

Toxicity will be evaluated using the National Cancer Institute Common Terminology Criteria for Adverse Events (NCI CTCAE v 4.03).

Dose Limiting Toxicity (DLT) is defined as any study-drug related adverse event, occurring during and the RAI therapy through to the end of pazopanib therapy at 8 weeks post RAI, which meets the following criteria:

- Any Grade ≥ 3 hematologic toxicity lasting ≥ 5 days, or febrile neutropenia
- Any Grade 4 hematologic toxicity.
- Any ≥ Grade 3 non-hematologic toxicity, except diarrhea, nausea or vomiting will only be considered dose limiting toxicity when ≥ Grade 3 toxicity occurs despite adequate anti-emetics or anti-diarrhea medications.
- Treatment delay due to toxicity lasting greater than 14 days since the last dose of pazopanib.
- Death.

"Study-drug related" refers to events that are possibly or probably related to the administration of pazopanib or the combined treatment regimen of pazopanib and RAI, and not clearly attributed to other causes (Toxicities during the administration of pazopanib alone during weeks 1 to 4 prior to RAI therapy will not be considered a DLT for dose escalation purposes as RAI will not have been administered in combination with pazopanib).

Patients will be replaced if:

- they are unable to complete at least 14 days of continuous dosing of pazopanib alone prior to combined RAI therapy (patients can continue if drug is held or dose reductions are required down to 600 mg po once daily prior to RAI therapy).

  OR
- they must discontinue the study prematurely due to disease-related complications (e.g., disease progression) or for any other reason(s).

  AND
- they have not experienced a DLT

Note: DLTs are adverse events that occur during the administration of and meet the specific criteria outlined above. The presence or absence of DLTs dictates whether cohorts must be expanded and whether or not dose escalation (enrollment of the next cohort) can occur. However, individual subjects who experience DLTs may nevertheless receive additional doses of pazopanib, following careful medical evaluation and appropriate treatment and/or a dose delay at the discretion of the Investigator.

5.13 Maximum Tolerated Dose

The maximum tolerated dose (MTD) will be the highest dose at which no more than one of six patients experience a DLT. The MTD level in this study will be expanded to enroll up to 6 evaluable patients. The MTD and general tolerability will be used in the determination of the RP2D.

5.14 Dose Modifications

In order to maintain dose-intensity and cumulative dose-delivery on this study, reasonable efforts will be made to minimize dose reduction and treatment delays as specified. Any patient whose treatment is delayed must be evaluated on a weekly basis until adequate hematologic and non-hematologic

parameters have been met. No intra-patient dose escalation is planned for this study. Patients should be monitored for study-drug related toxicity according to the procedures outlined in Appendix A.

The investigator should carefully assess all treatment-associated toxicities and, whenever possible, determine if they can reasonably be attributed to pazopanib alone, RAI, or the combination regimen. If appropriate, dose delays and/or adjustments should be restricted to the suspected causative agent. As previously noted, toxicity grades are defined using the NCI CTCAE version 4.03.

- In general, no more than two dose reduction is allowed for hematological or non-hematological toxicities of pazopanib (i.e. any patient who has had 2 dose reductions and who experiences a toxicity that would cause a third dose reduction should be discontinued from the study).
- Any patient who requires a dose reduction of pazopanib will continue to receive the reduced dose for the remainder of the study.
- No dose escalations are allowed in this study.

5.14.1 Pazopanib Dose Modifications

Refer to the Pazopanib label for current recommendations regarding dose delays and reductions.

As a general rule, if dose reduction of pazopanib is necessary, the dose should be reduced stepwise by 200 mg at each step, and the subject should be monitored for approximately 10 to 14 days at each dose level. If toxicity does not abate during this monitoring time, the IP may need to be interrupted and/or the dose further decreased with continued monitoring for an additional 10-14 days at each dose level, and so on.

If a subject's treatment has been interrupted for more than 14 days, the Investigator must contact the NCCN to review the subject's condition in order to resume the treatment. Patients that require dose reductions after 400 mg po once daily dosing of pazopanib should be taken off study.

Dose Interruptions/Modifications for Specific, Non-liver Related, Toxicities
Recommendations for investigational product dose interruptions/modifications in case of specific treatment-emergent AEs are provided in Table below. Management of Diarrhea and Nausea and Vomiting will be described in Appendix H

Table 3 Dose Modification Algorithms for Potential Treatment-Related Adverse Events

AE Terms & Descriptions	Dose Modification Algorithms
Hypertension	
(A). Asymptomatic and persistent SBP of ≥140 and <170 mmHg, or DBP ≥ 90 and < 110 mmHg, or a clinically significant increase in DBP of 20 mmHg (but still below 110 mmHg).	Step 1. Continue pazopanib at the current dose.<br>Step 2. Adjust current or initiate new antihypertensive medication(s).<br>Step 3. Titrate antihypertensive medication(s) during next 2 weeks as indicated to achieve well-controlled[a] blood pressure (BP). If BP is not well-controlled within 2 weeks, consider referral to a specialist and go to scenario (B).
(B). Asymptomatic SBP ≥ 170 mmHg, or DBP ≥ 110 mmHg, or failure to achieve well-controlled BP within 2 weeks in scenario (A).	Step 1.Consider reducing or interrupting IP, as clinically indicated.<br>Step 2. Adjust current or initiate new antihypertensive medication(s).<br>Step 3. Titrate antihypertensive medication(s) during next 2 weeks as indicated to

AE Terms & Descriptions	Dose Modification Algorithms
	achieve well-controlled BP.<br>Step 4. Once BP is well-controlled, restart pazopanib dose-reduced by 200 mg if IP was interrupted.
(C). Symptomatic hypertension or recurring SBP ≥ 170 mmHg, or DBP ≥ 110 mmHg, despite modification of antihypertensive medication(s)	Step 1. Interrupt IP<br>Step 2. Adjust current or initiate new antihypertensive medication(s).<br>Step 3. Titrate antihypertensive medication(s) during next 2 weeks as indicated to achieve well-controlled BP. Referral to a specialist for further evaluation and follow-up is also recommended.<br>Step 4. Once BP is well-controlled, restart pazopanib dose-reduced by 200 mg.
(D). Refractory hypertension unresponsive to above interventions.	Discontinue pazopanib and continue follow-up per protocol.
Prolongation of QTc Interval: If the QTc is prolonged, the ECG should be manually read to ensure accuracy of the reading. The values below refer to manually-read ECGs. REFER READER TO ECG SECTION IN PROTOCOL	
QTc ≥ 480 < 500 msec	Continue pazopanib; monitor as clinically indicated.
QTc ≥500 msec	Discontinue pazopanib and continue follow-up per protocol.
Proteinuria	
UPC <3	Continue pazopanib at the current dose; monitor as clinically indicated
UPC ≥ 3 or 24-h urine protein ≥ 3g	Step 1. Interrupt pazopanib.<br>Step 2. Weekly UPC or 24-hr urine protein monitoring until UPC is < 3 or 24-hr urine protein is < 3 grams. Then restart pazopanib dose-reduced by 200 mg.<br>Step 3. If UPC ≥ 3 or 24-h urine protein ≥ 3g recurs, repeat steps 1 and 2.<br>Step 4. If UPC ≥ 3 or 24-hr urine protein ≥ 3 recurs and the pazopanib dose can no longer be reduced, discontinue pazopanib and continue follow-up per protocol.
Hemorrhage /Bleeding: Investigate and document underlying etiology of the bleeding	
Grade 1	For hemoptysis, interrupt pazopanib and contact NCCN to discuss whether further treatment with pazopanib is appropriate.<br>For other Grade I hemorrhage/bleeding events, continue pazopanib at the current dose; monitor as clinically indicated.
Grade 2	Step 1. If pulmonary or GI bleed (other than hemorrhoidal bleeding), discontinue IP and continue follow-up per protocol. Otherwise, interrupt IP until the AE resolved to ≤ Grade 1.<br>Step 2. Restart pazopanib; consider reducing dose and monitor as clinically indicated.
Grade 3 or 4, or<br>Recurrent ≥ Grade 2 event after dose interruption/reduction.	Discontinue pazopanib and continue with follow-up per protocol.

AE Terms & Descriptions	Dose Modification Algorithms
Venous Thrombosis (DVT, PE)	
Grade 2	Continue pazopanib at the current dose; monitor as clinically indicated
Grade 3	Step 1. Interrupt pazopanib.<br>Step 2. Initiate and monitor anticoagulation as clinically indicated.<br>Step 3. Resume pazopanib same dose, only if all of the following criteria are met:<br>• The subject must have been treated with anticoagulant at the desired level of anticoagulation for at least one week.<br>• No Grade 3 or 4 or clinically significant Grade 2, hemorrhagic events have occurred while on anticoagulation treatment.<br>Subject should be monitored as clinically indicated during anticoagulation treatment and after resuming study treatment. When treating with warfarin, international normalized ratio (INR) should be monitored within three to five days after any change in pazopanib dosing (eg, re-initiating, escalating/de-escalating, or discontinuing pazopanib), and then at least weekly until the INR is stable. The dose of warfarin (or its derivatives) may need to be adjusted to maintain the desired level of anticoagulation
Grade 4 and/or PE	Discontinue pazopanib and continue follow-up per protocol.
Arterial Thrombosis/Ischemia	
Any Grade	Discontinue pazopanib and continue follow-up per protocol.
Thrombocytopenia: Investigate and document underlying cause	
Grade 1 or 2	Continue pazopanib with current dose; monitor as clinically indicated.
Grade 3 or 4	Step 1. Interrupt pazopanib until toxicity resolves to ≤ Grade 2.<br>Step 2. Restart pazopanib, dose-reduced by 200 mg and monitor as clinically indicated.<br>If no recovery to ≤ Grade 2 or recurrent Grade 3 or 4 thrombocytopenia, discontinue pazopanib and follow-up per protocol.
Anemia: No specific dose reduction rules are indicated for anemia unless due to hemorrhage or bleeding as noted above.	
Palmar-plantar Erythrodysesthesia Syndrome	
Grade 1<br>Minimal skin changes or dermatitis without pain<br>(erythema, oedema, hyperkeratosis)	1. Continue pazopanib at present dose
Grade 2<br>Skin changes with pain; limiting instrumental activities of daily living (ADLs) (peeling, blisters, oedema, bleed, hyperkeratosis)	1. Hold pazopanib<br>2. Treat as clinically appropriate<br>3. Upon resolution to Level 1 or better restart pazopanib with a dose reduction to 400 mg<br>4. If recurrent consider discontinuation

AE Terms & Descriptions	Dose Modification Algorithms
Grade 3<br>Severe skin changes with pain and limiting self care ADLs	1. Discontinue pazopanib
Other Clinically Significant Adverse Events[b]	
Grade 1	Continue pazopanib; monitor as clinically indicated.
Grade 2 or 3, if clinically significant	Step 1. Interrupt pazopanib until toxicity resolves to ≤ Grade 1.<br>Step 2. Restart pazopanib dose-reduced by 200 mg and monitor as clinically indicated.
Grade 4	Discontinue pazopanib and continue follow-up per protocol.

a. Well-controlled BP defined as SBP <140 mmHg and DBP <90 mmHg.
b. AEs are graded according to NCI Common Terminology Criteria for Adverse Events v4.03 (NCI CTCAE v4.03)
Abbreviations: BP, blood pressure; IP, investigational product.

Dose Interruptions/Modifications for Hepatotoxicity

Recommendations for pazopanib dose interruptions/modifications in case of liver-related treatment-emergent AEs are provided in Table 4. As a general rule, since many subjects are taking multiple concurrent medications, it is critical to (a) do a thorough evaluation of the subject's concurrent medications, and (b) identify and discontinue those with known hepatotoxicity and replace with a non-hepatotoxic equivalent for the same indication if necessary. Liver dysfunction must be fully evaluated even if clinical signs and symptoms indicate progression of liver tumor lesions. Imaging studies may be obtained to document potential progression of malignancy.

Table 4 Guidelines for Management of Treatment Emergent Hepatotoxicity

Event	Dose Modification Algorithms
(A). ALT of ≤ 3.0 x ULN	Continue pazopanib at current dose with full panel LFTs monitored as per protocol.
(B). ALT >3.0 x ULN to ≤ 8.0 x ULN without bilirubin elevation (defined as total bilirubin[b] < 2.0 x ULN or direct bilirubin ≤ 35%) and without hypersensitivity symptoms (e.g., fever, rash)	Liver Event Monitoring Criteria:<br>(1) Continue pazopanib at current dose levels.<br>(2) Monitor subject closely for clinical signs and symptoms; perform full panel LFTs weekly or more frequently if clinically indicated until ALT/AST is reduced to Grade 1.
(C). ALT > 8.0 x ULN without bilirubin elevation (defined as total bilirubin < 2.0 x ULN or direct bilirubin ≤ 35%) and without hypersensitivity symptoms (e.g., fever, rash)	1st occurrence – Liver Event Interruption CriteriaError! Reference source not found.:<br>(1) Interrupt pazopanib until toxicity resolves to ≤Grade 1 or baseline. Report the event to GSK as an SAE within 24 hours of learning of its occurrence . Make every reasonable attempt to have subjects return to the clinic within 24 to 72 hours for repeat liver chemistries and liver event follow up assessments.<br>(2) Liver imaging and other laboratory investigations should be considered as clinically appropriate.<br>(3) Monitor subject closely for clinical signs and symptoms; perform full panel LFTs weekly or more frequently if clinically indicated until ALT/AST is reduced to Grade 1.<br>(4) If the subject is benefiting from the study treatment, contact NCCN for possible re-challenge. Re-treatment may be considered if ALL following criteria are met:<br>- ALT/AST reduced to Grade 1<br>- Total bilirubin <1.5 x ULN or direct bilirubin ≤35%<br>- No hypersensitivity signs or symptoms<br>- Subject is benefiting from therapy.<br>Recurrence – Liver Event Stopping Criteria:<br>Discontinue pazopanib permanently and monitor subject closely for clinical signs and symptoms; perform full panel LFTs weekly or more frequently if clinically indicated until ALT/AST is reduced to Grade 1.
(D). ALT >3.0 x ULN with concomitant elevation in bilirubin[b] (defined as total bilirubin ≥ 2.0 x ULN; with direct bilirubin > 35%) or with hypersensitivity symptoms (e.g., fever, rash).	Liver Event Stopping CriteriaError! Reference source not found.:<br>(1) Discontinue pazopanib immediately, report the event to GSK as an SAE within 24 hours of learning of its occurrence. Make every reasonable attempt to have subjects return to the clinic within 24 hours for repeat liver chemistries and liver event follow up assessments.<br>(2) Consult a gastroenterologist / hepatologist, and perform the following assessments to identify potential co-factors:<br>- Eosinophil count<br>- Viral serology for hepatitis A, B, C and E, cytomegalovirus, Epstein-Barr virus (IgM antibody, heterophile antibody, or monospot testing)<br>- Anti-nuclear antibody, anti-smooth muscle antibody, Type 1 anti-liver kidney microsomal antibodies.<br>- Serum creatinine phosphokinase for possible muscle injury caused LFT elevation<br>- Liver imaging<br>-Consider toxicological blood screen for possible contributing chemical/medical entities<br>(3) Monitor subject closely for clinical signs and symptoms; record the appearance or worsening of clinical symptoms of hepatitis, or hypersensitivity, such as fatigue, nausea, vomiting, right upper quadrant pain or tenderness, fever rash or eosinophilia as relevant on the AE report form. Perform full panel LFTs weekly or more frequently if clinically indicated until LFTs are reduced to Grade 1.
For isolated total bilirubin[b] elevation without concurrent ALT increases (defined as ALT <3 X ULN).	(1) Isolated hyperbilirubinemia (i.e., in the absence of elevated ALT or other signs/symptoms of liver injury) does not require dose modification. Pazopanib inhibits UGT1A1 and OATP1B1, which can cause elevation of indirect (unconjugated) bilirubin in the absence of liver injury..<br>(2) If bilirubin is >1.5 x ULN in the absence of ALT elevation, fractionation of bilirubin elevation should be performed. If bilirubin is >35% direct (conjugated), further evaluation for underlying cause of cholestasis should be performed.

a. Full panel LFTs include: AST, ALT, alkaline phosphatase, and total bilirubin. Coagulation tests should be performed as clinically indicated.

b. Serum bilirubin fractionation should be performed if testing is available. If testing is unavailable and a subject meets the criterion of total bilirubin >1.5 x ULN, then the event should be promptly reported as an SAE.

Abbreviations: ALT alanine aminotransferase; AST aspartate aminotransferase; IP investigational product; LFT liver function tests; SAE serious adverse event; ULN upper limit of normal

5.14.2 TREATMENT OF INVESTIGATIONAL PRODUCT OVERDOSE

No maximum tolerated dose (MTD) was reached in the dose escalation study of pazopanib administered as a single agent at repeated doses of up to 2000 mg/day (Study VEG10003). Systemic exposure to pazopanib at steady-state appeared to plateau at doses greater than 800 mg once daily. Increases in the daily pazopanib dose above 800 mg in the fasted state resulted in a small or no increase in mean systemic exposure to pazopanib.

In the event of pazopanib overdose (defined as administration of more than the protocol-specified dose), the investigator should contact GSK. Decisions regarding pazopanib dose modifications or interruptions will be made by the investigator in consultation with GSK based on the clinical evaluation of the subject. Following an overdose, additional monitoring of the subject for AEs/SAEs and laboratory abnormalities should be considered.

6.0 DATA ANALYSIS/STATISTICAL METHODS

Sample Size Determination: The number of patients to be enrolled in the study will depend upon the observed safety profile, which will determine the number of patients per dose level and the number of dose escalations. It is anticipated that a total of approximately 12 to 15 patients (maximum of 18 patients) will be enrolled in this study with an estimated annual accrual of 12. The study will take place at a single center.

The operating characteristics of this study design are shown in Table 5, which provides the probability of escalation to the next higher dose for each underlying true DLT rate. For example, for a toxicity that occurs in 5% of subjects, there is a greater than 95% probability of escalating. Conversely, for a common toxicity that occurs with a rate of 70%, the probability of escalating is <5%.

Table 5: Probability of Escalation to the Next Dose for Each True Underlying DLT Rate at a Dose Level

True Underlying DLT Rate	5%	10%	20%	30%	40%	50%	60 %	70%	80%	90%
Probability of Escalating Dose	0.97	0.91	0.71	0.49	0.31	0.17	0.0 8	0.03	0.01	0.001

Table 6 shows the probability of failing to observe toxicity in a sample size of 3 or 6 patients given various true underlying toxicity rates. For example, with 6 patients, the probability of failing to observe toxicity occurring at least 40% of the time is less than 5%.

Table 6: Probability of Failing to Observe True Underlying DLT Rate at a Dose Level

True Underlying DLT Rate	5%	10%	20%	30%	40%	50%	60%	70%	80%	90%
Probability of Failing to	0.86	0.73	0.51	0.34	0.22	0.13	0.064	0.027	0.008	0.001

Observe Toxicity, N=3										
Probability of Failing to Observe Toxicity, N=6	0.74	0.53	0.26	0.12	0.047	0.016	0.0041	<0.001	<0.001	<0.001

Data Analysis

The study population for toxicity analyses will include all patients enrolled in the study who receive at least one dose of study medication pazopanib. Efficacy will be determined based on patients who receive the combination of pazopanib and RAI. Due to the exploratory nature of this study, no confirmatory inferential analyses are planned, and no imputation for missing data will be done. Descriptive statistics (such as means, medians, standard deviations and ranges for continuous data and percentages for categorical data) will be used to summarize patient characteristics, treatment administration/compliance, efficacy, safety, and correlative study data. Data will also be displayed graphically, where appropriate.

6.1 Analysis of Primary Endpoint

The primary end point of this phase I study is to assess the toxicity and the occurrence of dose limiting toxicity (DLT) when pazopanib is given in conjunction with radioiodine to establish the MTD and RP2D in combination. Toxicities will be classified by NCI common toxicity criteria. For each cohort DLT's will be summarized by category (hematologic and non-hematologic) and by MedDRA preferred term.

The period for determination of dose-limiting toxicity (DLT) for the purposes of dose escalation and the MTD will be determined from the first 8 weeks post radioactive iodine administration; whereas DLT, dose modifications, and other toxicities will be assessed over all courses when determining the optimal dose for phase II evaluation. Descriptive statistics will be calculated for all variables and responses; continuous data will be expressed as their mean ± standard deviation, median and range, and categorical data will be listed by frequency of occurrence and proportion of total (with 95% confidence intervals) for all enrolled patients and by dose cohort.

6.2 Analysis of Secondary Endpoints

Secondary endpoints include post-pazopanib treatment tumor response (RECIST criteria), and TTP with respect to censored baseline imaging and suppressed thyroglobulins at week 14+. Tumor response and TTP will be compared to responses and duration of TTP after last prior historical RAI treatment.

Objective Response:

For each cohort and tumor type, the best response (CR, PR, SD or PD according to RECIST criteria) for each patient with measurable disease who received pazopanib in combination with RAI will be listed. Progression-free survival will be determined in days or week and waterfall plots and graphical data will be provided where suitable.

Analysis of Other Endpoints

Analysis of Clinical Labs: Listing tables will be prepared for each laboratory measure, and will be structured to permit review of the data by patient as they progress on treatment. The tables will list the schedule, day and cycle of treatment, pazopanib dose, RAI dose and associated NCI CTCAE toxicity grade.

Summary tables and graphic displays, as appropriate, will be prepared to examine the distribution of these toxicities per cycle. Graphic displays and shift tables may be provided to illustrate the results over time on study. Assessment of cumulative toxicities may be made.

6.3 Analysis of Other Endpoints

Tertiary endpoints involve assessing increased radioiodine uptake, retention (through comparison of radioiodine scans in prior historical studies) and correlative effects of pazopanib on tumor blood flow and response in WDTC (assessed by dynamic FDG-PET). will be predominantly descriptive with graphical information where available.

7.0 ASSESSMENTS

Safety Assessment and Determination of Dose Level for Subsequent Patient Cohorts. Subjects will be required to have the following assessments, as specified in the Schedule of Events (Appendix A). These assessments may be performed more frequently than specified if clinically indicated.

7.1 Laboratory Safety Assessments

Comprehensive blood work: Hematology and blood chemistry will be drawn at the time points described in the Schedule of Events (Appendix A) and analyzed at local laboratories. Investigators may have additional blood tests performed for the purpose of planning treatment administration, dose modification, or following adverse events.

Pregnancy test: Serum or urine pregnancy test for women of childbearing potential will be performed by a local laboratory. A screening serum β-HCG pregnancy test is mandatory for all women of child-bearing potential and should be done within 2 weeks prior to the first dose of study medication. Thereafter, the serum pregnancy test only needs to be repeated if clinically indicated or as required by local regulation.

7.2 Other Safety Assessments

Physical examination: At screening, a complete medical history and physical examination—including height (baseline only), body weight, vital signs, and examination of relevant body organ systems, concomitant treatments, performance status will be performed and repeated at subsequent visits, according to Appendix A. Any new or worsening medical condition from the baseline level (pre-dose on Day 1) should be recorded in the AE or SAE eCRF.

Specific Additional Safety Assessments

Vital Signs and Blood Pressure Monitoring
Hypertension is a common drug-related AE with pazopanib; therefore, frequent blood pressure monitoring is mandatory.

ECOG PS
If subjects discontinue study treatment without disease progression (e.g., withdrawal of study treatment due to unacceptable toxicity), continue the assessments of ECOG PS in accordance with the disease assessments until subjects experience disease progression.

12-Lead Electrocardiogram
In clinical studies with pazopanib, events of QT prolongation have occurred. A 12-lead ECG will be obtained according to Appendix A.

Screening/Baseline QTc

- If QTc interval is > 480 msecs, then 2 additional ECGs should be obtained over a brief period of time (e.g., within 15-20 minutes) to confirm the abnormality.
- The average QTc interval will be determined from the 3 ECG tracings by manual evaluation and will be used to determine if the subject will be excluded from the study.
- If the average QTc interval is >480 msec, then the subject is not eligible to participate in the study.

During Treatment QTc

- If the average QTc is less ≤ 500 msec, the subject may continue therapy.
- If a QTc >500 msec is noted on a scheduled or unscheduled ECG, then 2 additional ECGs should be obtained over a brief period of time (e.g., within 15-20 minutes) to confirm the abnormality.
  - Each ECG tracing should be evaluated manually to obtain RR and QT intervals for QTc calculation.
  - The average QTc will be determined from the 3 ECG tracings by manual evaluation and will be used to determine appropriate next steps. A cardiologist should be consulted, if necessary, to determine the diagnosis of QT prolongation.
- If the average QTc is >500 msec, the following steps should be taken:
  - Study treatment should be interrupted immediately.
  - Electrolytes, particularly potassium and magnesium, should be checked and corrected if abnormal.
  - Concomitant medications with a potential for QTc interval prolongation should be discontinued if clinically appropriate.

- A cardiologist should be consulted to assist with the management of the subject if clinically appropriate.
- The subject should be treated appropriately for QTc prolongation and monitored until resolution is documented by a repeat ECG with QTc interval returning to ≤ 480 msec.

- If the QTc prolongation > 500 msec is clearly and causally associated with an underlying situation that is clearly reversible (e.g., a subject with severe diarrhea and hypokalemia with QTc prolongation that resolves once the diarrhea improves and potassium is corrected), then the subject may restart study drug once the underlying situation has been corrected (e.g., electrolytes supplemented) and the QTc interval prolongation has resolved.

- If the QTc prolongation > 500 msec is not clearly and causally associated with an underlying situation that is clearly reversible, then the subject should have study drug permanently discontinued and be withdrawn from the study.

Safety Assessments upon Discontinuation of Study Treatment

Subjects should have the following safety assessments performed upon discontinuation of study treatment: physical examination and vital signs, ECOG PS, clinical chemistry, hematology, and UPC. Laboratory tests do not need to be repeated if less than 4 weeks have elapsed since most recent assessments. Coagulation tests and ECG should be performed if clinically indicated. The date and reason for discontinuation of study treatment must be recorded clearly in the Study Treatment Discontinuation eCRF.

7.3. Adverse Events

Assessment of adverse events will include type, incidence, severity (graded by the National Cancer Institute [NCI] Common Terminology Criteria for Adverse Events [CTCAE], Version 4.03), timing, seriousness, and relatedness; and laboratory abnormalities.

Patients who receive 1 or more doses of pazopanib will be evaluable for toxicity.

7.3.1 Adverse Event Reporting

In the event of an adverse event the first concern will be for the safety of the subject. The adverse event recording period will start on the first day of study treatment and end 28 days (+/- 3 days) of termination pazopanib dosing.

All adverse events will be reported for the 14 weeks of pazopanib therapy. Thereafter, only AEs which can reasonably be attributed to pazopanib and/or RAI will be reported.

7.3.2 Adverse Event Causality

The Investigator will use the following definitions to assess the relationship of the adverse event to the use of study drug:

Related - An adverse event has a strong temporal relationship to study drug, recurs on re-challenge or is known to be an effect of the study drug. Another reasonable etiology either doesn't exist or is unlikely.

Possibly Related - An adverse event has a strong temporal relationship to the study drug and an alternative etiology is either equally or less likely when compared to the potential relationship to study drug.

Not Related - An adverse event is due to an underlying or concurrent illness or effect of another drug and is not related to the study drug (e.g., has little or no temporal relationship to study drug or has a much more likely alternative etiology).

7.4 Serious Adverse Event Reporting

Investigators are required to report to the Sponsor-Investigator ANY serious adverse event (SAE) as soon as possible.

An SAE is any sign, symptom or medical condition that emerges during treatment or during a post-treatment follow-up period that (1) was not present at the start of treatment and it is not a chronic condition that is part of the patient's medical history, OR (2) was present at the start of treatment or as part of the patient's medical history but worsened in severity and/or frequency during therapy, AND that meets any of the following regulatory serious criteria:

- Results in death
- Is life-threatening
- Requires or prolongs inpatient hospitalization (planned hospitalizations (e.g. radiation isolation) are not considered an SAE
- Is disabling or incapacitating
- Is a congenital anomaly/birth defect
- Is medically significant or requires medical or surgical intervention to prevent one of the outcomes listed above.

Serious adverse events require immediate notification to the following party below, beginning from the time of the first dose of investigational product, pazopanib, through and including 30 calendar days after the last administration of pazopanib. All emergent SAEs should be recorded on a MedWatch 3500A Form and communicated to:

Laura QM Chow, MD
Division of Medical Oncology, Box 358081
University of Washington (SCCA)
825 Eastlake Avenue E, MS:G4-940
Seattle WA, 98109-1023
Fax 206-288-1435, phone 206-288-6968
lchow@seattlecca.org

SAEs will be reported to the Cancer Consortium IRB in accordance with the AE reporting policy.

All SAEs that are unexpected (i.e., not in the current pazopanib Investigator Brochure) and considered related or possibly related to the use of the study drug must be reported to FDA within 15 calendar days, or within 7 calendar days if the SAE was fatal or life-threatening.

Additional Reporting to GSK and NCCN:

Any serious adverse events which occur during the clinical study or within 5 days of receiving the last dose of study medication, whether or not related to the study drug, must be reported by the investigator. In addition, any SAEs which occur as a result of protocol specific diagnostic procedures or interventions must also be reported.

**All serious adverse events must be reported by facsimile within 24 hours to GlaxoSmithKline.
MDC - Oncology Fax: (610) 675-2632**

For medical emergencies contact:
Toll Free Number: (800) 877-7074, ext. 7194
After Hours or Weekends: (800) 366-8900, ask for physician on call
GlaxoSmithKline UP4420
1250 S. Collegeville Road,
P.O. Box 5089
Collegeville, PA 19426-0989

And

NCCN via fax at 215-358-7699 or e-mailed to ORPReports@nccn.org

The SAE report should comprise a full written summary, detailing relevant aspects of the adverse events in question. Where applicable, information from relevant hospital case records and autopsy reports should be included. Follow-up information should be forwarded to GSK within 24 hours.

SAEs brought to the attention of the Investigator at any time after cessation of pazopanib and considered by the investigator to be related or possibly related to pazopanib must be reported to GSK if and when they occur. Additionally, in order to fulfill international reporting obligations, SAEs that are related to study participation (e.g., procedures, invasive tests, change from existing therapy) or are related to a concurrent medication will be collected and recorded from the time the subject consents to participate in the study until he/she is discharged

In addition, the Investigator will adhere to the safety reporting requirements and timelines described in the Clinical Trial Agreement with National Comprehensive Cancer Network (NCCN).

7.5 Efficacy Assessment

Evaluation of Disease Progression

During the course of the study, the Investigator will assess a subject's disease status based on the radiological assessment and clinical assessment, and will make clinical decisions on the subject's care based on medical judgment..

Disease response criteria will be determined as per RECIST criteria version1.1 (Appendix D)

8.0 REGULATORY, QUALITY AND ADMINISTRATIVE REQUIREMENTS

8.1 Source Data Verification

Monitoring of source documents will occur during the active treatment phase of the trial. Monitoring visits will be arranged by the Monitoring Program Coordinator at the Fred Hutchinson Cancer Research Center. The monitors are independent contractors and are external to the Cancer Consortium (University of Washington and Fred Hutchinson Cancer Research Center). Study monitors will perform ongoing source data verification to confirm that critical protocol data transcribed on the CRFs by authorized site personnel are accurate, complete, and verifiable from source documents. To facilitate source documentation verification, the investigator(s) and institution(s) must provide the Monitor direct

access to applicable source documents and reports for trial-related monitoring, audits, and IRB/EC review.

The investigational site must also allow inspection by applicable regulatory authorities.

8.2 Compliance with Laws and Regulations

The proposed study will be conducted according to the International Conference on Harmonization E6 Guideline for Good Clinical Practice (GCP) and the Declaration of Helsinki and the requirements of the Federal Regulations. Please refer to:

International Conference on Harmonization and GCP: http://www.fda.gov/oc/gcp/guidance.html

Declaration of Helsinki: http://www.fda.gov/oc/health/helsinki89.html

Code of Federal Regulations, Title 21:
http://www.accessdata.fda.gov/scripts/cdrh/cfdocs/cfcfr/CFRSearch.cfm]

8.3 Informed Consent

The informed consent documents must be signed and dated by the patient, or the patient's legally authorized representative, before his or her participation in the study. The case history for each patient shall document that informed consent was obtained prior to participation in the study. A copy of the informed consent documents must be provided to the patient or the patient's legally authorized representative.

8.4 Institutional Review Board

This protocol, the informed consent document, and relevant supporting information must be submitted to the IRB for review and must be approved before the study is initiated. In addition, any advertising materials must be approved by the IRB. The study will be conducted in accordance with applicable national and local health authority and IRB requirements.

The Principal Investigator is responsible for keeping the IRB apprised of the progress of the study and of any changes made to the protocol as deemed appropriate, but in any case the IRB must be updated at least once a year. In addition, the Principal Investigator is required to promptly notify the IRB of all adverse drug reactions that are both serious and unexpected. This generally refers to serious adverse events that are not already identified in the Investigator Brochure and that are considered possibly or probably related to the study drug by the Investigator.

8.5 Retention of Records

U.S. FDA regulations (21 CFR §312.62[c]) require that records and documents pertaining to the conduct of this study and the distribution of investigational drug—including CRFs, consent forms, laboratory test results, and medication inventory records—must be retained by the Principal Investigator for 5 years after marketing approval is received for pazopanib or for 5 years after all clinical and product development of pazopanib is discontinued and the applicable national and local health authorities are notified. The Principal Investigator must notify GSK/NCCN prior to the destruction of any records relating to this study.

8.6 Drug Accountability

GSK Pharmaceuticals will provide the Principal Investigator with adequate supplies of pazopanib for the study population and protocol requirements. Damaged supplies will be replaced. Drug supplies must be kept in an appropriate, secure area (e.g., locked pharmacy) and stored in accordance with the conditions specified in this protocol and on the investigational drug labels.

Drug supplies are to be used only in accordance with this protocol. Investigational drug may be administered only to eligible subjects who are enrolled in the study, and the Sponsor-Investigator is accountable for all used and unused investigational drug. Used and partially used study drug will be destroyed according to the standard practice of the Investigational Pharmacy at the University of Washington / Seattle Cancer Care Alliance. Upon written notification, the Principal Investigator will ship unused investigational drug according to instructions provided by GSK Pharmaceuticals, Inc. All material containing pazopanib will be treated and disposed of as hazardous waste in accordance with governing regulations.

Study drug accountability records should be maintained by the site in accordance with the regulations. A master drug log must be maintained of all pazopanib vials received, dispensed (including the lot number of the vials, the subject's ID number, the subject's initials, and the dates each vial is dispensed), and returned or destroyed. In addition, a subject-specific record of each vial administered, including the date and lot number of each vial, will be maintained with the case file for each subject. Any discrepancy in the drug distribution logs (master log and subject logs) must be explained in detail.

8.7 Premature Closure of the Study

This study may be prematurely terminated, if in the opinion of the Principal-Investigator, NCCN or GSK Pharmaceuticals Inc, there is sufficient reasonable cause. Written notification documenting the reason for study termination will be provided to the Principal Investigator, NCCN or GSK Pharmaceuticals Inc by the terminating party.

Circumstances that may warrant termination include, but are not limited to:

- Determination of unexpected, significant, or unacceptable risk to patients.
- Failure to enter patients at an acceptable rate.
- Insufficient adherence to protocol requirements.
- Insufficient complete and/or evaluable data.
- Plans to modify suspend or discontinue the development of the drug.

9.0 REFERENCES

1. Davies L, Welch HG: Thyroid cancer survival in the United States: observational data from 1973 to 2005. Arch Otolaryngol Head Neck Surg 136:440-4

2. Davies L, Welch HG: Epidemiology of head and neck cancer in the United States. Otolaryngol Head Neck Surg 135:451-7, 2006

3. Davies L, Welch HG: Increasing incidence of thyroid cancer in the United States, 1973-2002. Jama 295:2164-7, 2006

4. Jemal A, Siegel R, Xu J, et al: Cancer statistics, 2010. CA Cancer J Clin 60:277-300

5. Cooper DS, Doherty GM, Haugen BR, et al: Revised American Thyroid Association management guidelines for patients with thyroid nodules and differentiated thyroid cancer. Thyroid 19:1167-214, 2009

6. Burns WR, Zeiger MA: Differentiated thyroid cancer. Semin Oncol 37:557-66

7. Mazzaferri EL: An overview of the management of papillary and follicular thyroid carcinoma. Thyroid 9:421-7, 1999

8. Rutherford GC, Franc B, O'Connor A: Nuclear medicine in the assessment of differentiated thyroid cancer. Clin Radiol 63:453-63, 2008

9. Schlumberger M, Tubiana M, De Vathaire F, et al: Long-term results of treatment of 283 patients with lung and bone metastases from differentiated thyroid carcinoma. J Clin Endocrinol Metab 63:960-7, 1986

10. Durante C, Haddy N, Baudin E, et al: Long-term outcome of 444 patients with distant metastases from papillary and follicular thyroid carcinoma: benefits and limits of radioiodine therapy. J Clin Endocrinol Metab 91:2892-9, 2006

11. Maxon HR: Quantitative radioiodine therapy in the treatment of differentiated thyroid cancer. Q J Nucl Med 43:313-23, 1999

12. Maxon HR, 3rd, Smith HS: Radioiodine-131 in the diagnosis and treatment of metastatic well differentiated thyroid cancer. Endocrinol Metab Clin North Am 19:685-718, 1990

13. Wang W, Larson SM, Tuttle RM, et al: Resistance of [18f]-fluorodeoxyglucose-avid metastatic thyroid cancer lesions to treatment with high-dose radioactive iodine. Thyroid 11:1169-75, 2001

14. Bible KC, Suman VJ, Molina JR, et al: Efficacy of pazopanib in progressive, radioiodine-refractory, metastatic differentiated thyroid cancers: results of a phase 2 consortium study. Lancet Oncol 11:962-72

15. Cabanillas ME, Waguespack SG, Bronstein Y, et al: Treatment with tyrosine kinase inhibitors for patients with differentiated thyroid cancer: the M. D. Anderson experience. J Clin Endocrinol Metab 95:2588-95

16. Carr LL, Mankoff DA, Goulart BH, et al: Phase II study of daily sunitinib in FDG-PET-positive, iodine-refractory differentiated thyroid cancer and metastatic medullary carcinoma of the thyroid with functional imaging correlation. Clin Cancer Res 16:5260-8

17. Deshpande HA, Gettinger S, Sosa JA: Axitinib: The evidence of its potential in the treatment of advanced thyroid cancer. Core Evid 4:43-8

18. Deshpande HA, Gettinger SN, Sosa JA: Novel chemotherapy options for advanced thyroid tumors: small molecules offer great hope. Curr Opin Oncol 20:19-24, 2008

IRB Approved
Document Release Date: 10/22/2015

19. Duntas LH, Bernardini R: Sorafenib: rays of hope in thyroid cancer. Thyroid 20:1351-8

20. Gupta-Abramson V, Troxel AB, Nellore A, et al: Phase II trial of sorafenib in advanced thyroid cancer. J Clin Oncol 26:4714-9, 2008

21. Kloos RT, Ringel MD, Knopp MV, et al: Phase II trial of sorafenib in metastatic thyroid cancer. J Clin Oncol 27:1675-84, 2009

22. Schlumberger M: Kinase inhibitors for refractory thyroid cancers. Lancet Oncol 11:912-3

23. Sherman SI: Targeted therapy of thyroid cancer. Biochem Pharmacol 80:592-601

24. Sherman SI: Tyrosine kinase inhibitors and the thyroid. Best Pract Res Clin Endocrinol Metab 23:713-22, 2009

25. Lennard CM, Patel A, Wilson J, et al: Intensity of vascular endothelial growth factor expression is associated with increased risk of recurrence and decreased disease-free survival in papillary thyroid cancer. Surgery 129:552-8, 2001

26. Hsueh C, Lin JD, Wu IC, et al: Vascular endothelial growth factors and angiopoietins in presentations and prognosis of papillary thyroid carcinoma. J Surg Oncol 103:395-9

27. Schutz FA, Choueiri TK, Sternberg CN: Pazopanib: Clinical development of a potent anti-angiogenic drug. Crit Rev Oncol Hematol 77:163-71

28. Hurwitz HI, Dowlati A, Saini S, et al: Phase I trial of pazopanib in patients with advanced cancer. Clin Cancer Res 15:4220-7, 2009

29. Hyer SL, Newbold K, Harmer CL: Early and late toxicity of radioiodine therapy: detection and management. Endocr Pract 16:1064-70

30. Gao YC, Lu HK: Outcome after high-dose radioiodine therapy for advanced differentiated thyroid carcinoma in childhood. Endocr Res 34:121-9, 2009

31. Kohandel M, Kardar M, Milosevic M, et al: Dynamics of tumor growth and combination of anti-angiogenic and cytotoxic therapies. Phys Med Biol 52:3665-77, 2007

32. Pietras K, Hanahan D: A multitargeted, metronomic, and maximum-tolerated dose "chemo-switch" regimen is antiangiogenic, producing objective responses and survival benefit in a mouse model of cancer. J Clin Oncol 23:939-52, 2005

33. Duda DG: Antiangiogenesis and drug delivery to tumors: bench to bedside and back. Cancer Res 66:3967-70, 2006

34. Senan S, Smit EF: Design of clinical trials of radiation combined with antiangiogenic therapy. Oncologist 12:465-77, 2007

35. Hallahan DE, Chen AY, Teng M, et al: Drug-radiation interactions in tumor blood vessels. Oncology (Williston Park) 13:71-7, 1999

36. Liu YY, Stokkel MP, Morreau HA, et al: Radioiodine therapy after pretreatment with bexarotene for metastases of differentiated thyroid carcinoma. Clin Endocrinol (Oxf) 68:605-9, 2008

37. Gershengorn MC, Izumi M, Robbins J: Use of lithium as an adjunct to radioiodine therapy of thyroid carcinoma. J Clin Endocrinol Metab 42:105-11, 1976

38. Liu YY, van der Pluijm G, Karperien M, et al: Lithium as adjuvant to radioiodine therapy in differentiated thyroid carcinoma: clinical and in vitro studies. Clin Endocrinol (Oxf) 64:617-24, 2006

39. Pons F, Carrio I, Estorch M, et al: Lithium as an adjuvant of iodine-131 uptake when treating patients with well-differentiated thyroid carcinoma. Clin Nucl Med 12:644-7, 1987

Printed on 12/7/2015

40. Hoftijzer H, Heemstra KA, Morreau H, et al: Beneficial effects of sorafenib on tumor progression, but not on radioiodine uptake, in patients with differentiated thyroid carcinoma. Eur J Endocrinol 161:923-31, 2009

41. Tseng J, Dunnwald LK, Schubert EK, et al: 18F-FDG kinetics in locally advanced breast cancer: correlation with tumor blood flow and changes in response to neoadjuvant chemotherapy. J Nucl Med 45:1829-37, 2004

42. Cai W, Chen X: Multimodality imaging of vascular endothelial growth factor and vascular endothelial growth factor receptor expression. Front Biosci 12:4267-79, 2007

43. Weber F: PET scans and traumatic brain injuries: a brief comment. Aviat Space Environ Med 77:1083, 2006

44. Weber WA: Positron emission tomography as an imaging biomarker. J Clin Oncol 24:3282-92, 2006

45. Doot RK, Dunnwald LK, Schubert EK, et al: Dynamic and static approaches to quantifying 18F-FDG uptake for measuring cancer response to therapy, including the effect of granulocyte CSF. J Nucl Med 48:920-5, 2007

46. Dunnwald LK, Gralow JR, Ellis GK, et al: Tumor metabolism and blood flow changes by positron emission tomography: relation to survival in patients treated with neoadjuvant chemotherapy for locally advanced breast cancer. J Clin Oncol 26:4449-57, 2008

47. Pandite L. AC, Ball H., et. al. : Investigator's Brochure for pazopanib (GW786034) for Oncology and Ophthalmic Indications (Version 08, RR2002/00017/10). Collegeville, PA, Glaxo Smith Kline Oncology Worldwide Development, February 28, 2011

48. Kumar R, Knick VB, Rudolph SK, et al: Pharmacokinetic-pharmacodynamic correlation from mouse to human with pazopanib, a multikinase angiogenesis inhibitor with potent antitumor and antiangiogenic activity. Mol Cancer Ther 6:2012-21, 2007

49. Sonpavde G, Hutson TE: Pazopanib: a novel multitargeted tyrosine kinase inhibitor. Curr Oncol Rep 9:115-9, 2007

50. Schutz FA, Choueiri TK, Sternberg CN: Pazopanib: Clinical development of a potent anti-angiogenic drug. Crit Rev Oncol Hematol

51. Sternberg CN: Pazopanib in renal cell carcinoma. Clin Adv Hematol Oncol 8:232-3

52. Tuttle R: Radioiodine Ttreatment of Differentiated Thyroid Cancer. Up To Date. www.uptodate.com, Waltham, MA, USA. updated February 17, 2011, Ross DS and Mulder JE (editors) 2011

53. Maxon HR, 3rd, Englaro EE, Thomas SR, et al: Radioiodine-131 therapy for well-differentiated thyroid cancer--a quantitative radiation dosimetric approach: outcome and validation in 85 patients. J Nucl Med 33:1132-6, 1992

54. Maxon HR, Thomas SR, Hertzberg VS, et al: Relation between effective radiation dose and outcome of radioiodine therapy for thyroid cancer. N Engl J Med 309:937-41, 1983

55. Sisson JC, Giordano TJ, Jamadar DA, et al: ^{131}-I treatment of micronodular pulmonary metastases from papillary thyroid carcinoma. Cancer 78:2184-92, 1996

56. Sisson JC, Jamadar DA, Kazerooni EA, et al: Treatment of micronodular lung metastases of papillary thyroid cancer: are the tumors too small for effective irradiation from radioiodine? Thyroid 8:215-21, 1998

IRB Approved
Document Release Date: 10/22/2015

57. Benua RS, Cicale NR, Sonenberg M, et al: The relation of radioiodine dosimetry to results and complications in the treatment of metastatic thyroid cancer. Am J Roentgenol Radium Ther Nucl Med 87:171-82, 1962

58. Billemont B, Medioni J, Taillade L, et al: Blood glucose levels in patients with metastatic renal cell carcinoma treated with sunitinib. Br J Cancer 99:1380-2, 2008

Printed on 12/7/2015

10.0 SUPPLEMENTS AND APPENDICES

7529

10.1 Appendix A: Schedule of Protocol Activities and Assessments

	**Protocol Activity**	**Screen Period**	**Pazopanib monotherapy weeks 1 to 4**					**Pazopanib with RAI weeks 5 to 7**			**Pazopanib monotherapy post RAI Weeks 8 to 14**							**EOS**	**28 day safety visit**	**LTFU (q 3-6 mo)**
		Week	1	2	3	4	Re-assess*	5	6	7	8	9	10	11	12	13	14			
		Day	1	8	15	22	22 to 28	29	36	43	50	57	64	71	78	84	91			
	Informed Consent [1]	X																		
	Medical History; Demographics	X																		
Baseline	BSA	X	(X)				X	(X)												
Baseline	Baseline Signs & Symptoms [2]	(X)	(X)				X													
Baseline	12-lead ECG [3]	X	(X)				X						X					(X)		
Labs[4]	Hematology [5]	X	(X)	(X)	(X)	(X)	X	(X)		X	X		(X)		X		(X)	X	(X)	
Labs[4]	Chemistry [6]	X	(X)	(X)	(X)	(X)	X	(X)		X	X		(X)		X		(X)	X	(X)	
Labs[4]	INR/aPTT	X																		
Labs[4]	UPC [7]	X	(X)				X						X					X	(X)	
Labs[4]	Pregnancy Test [8]	X																		
Labs[4]	Suppressed thyroglobulin levels, TSH, free T3 and free T4	X	(X)														X			X
Rx	Pazopanib [9]		X	X	X	X	X	X	X	X	X	X	X	X	X	X	X			
Rx	Radioiodine I-^{131} [10]								X											
Correlative Studies	Dynamic PET scans [11]	X					X													
Assessments	Vital Signs; PE [12]	X	X	X	X		X	(X)	X		X		X	X	X	X	X	X	X	
Assessments	Adverse Events [13]	X	X	X	X	X	X	(X)	X	(x)	X		X	X	X	X	X	X	X	
Assessments	Concomitant Meds [14]	X																		

Tumor Imaging [15]	X[16]	X	X	X

*** Patients will be assessed for pazopanib toxicities in this period. If toxicities deemed intolerable or severe toxicities (≥ Grade 3) due pazopanib occur during pazopanib monotherapy, drug may be held and patients may have a dose reduction down to 600 mg po daily, if a minimum of 14 days of pazopanib therapy cannot be administered prior to RAI treatment, patients will be taken off study, considered unevaluable and replaced.**

(X) Optional; see endnote text

---

[1] **Informed Consent**: Must be obtained prior to undergoing any study procedure and may occur up to 28 days prior to treatment.

[2] **Baseline Signs and Symptoms**: Patients will be asked about any signs or symptoms experienced within the past 14 days prior to Day 1. Baseline Assessment will be collected once for each patient, and in Assessment Period (within 7 days prior to Day 1)

[3] **ECG**: 12-lead ECGs should be performed in the morning and should be collected before or more than 30 minutes after needle sticks (e.g. phlebotomy and intravenous access procedures). If the mean QTc interval is prolonged (>500 msec) or major abnormalities are present, then the ECGs should be reviewed by a Cardiologist at the clinical site for confirmation.

[4] **Laboratory Studies**: Clinical samples will be analyzed by local laboratories.

[5] **Hematology**: WBC with differential, hemoglobin, and platelet count. If CBC is drawn during Screening within 7 days of Study Day 1, it is not necessary to repeat this test on Day 1 of pazopanib therapy. Counts should be reviewed before RAI treatment, and therapy should be held if ANC <1500/μL and platelets <90,000.

[6] **Blood Chemistry**: Total bilirubin, AST, ALT, alkaline phosphatase, albumin, sodium, potassium, chloride, calcium, BUN, creatinine and glucose. If drawn during Screening (within 7 days of pazopanib Day 1 treatment , or within 7 days of week 5 therapy, it is not necessary to repeat these studies .

[7] **UPC**: Urine Protein Creatinine Ratio

[8] **Pregnancy Test (serum)**: Women of reproductive potential must be tested within 21 days starting study treatment.

[9] **Pazopanib**: Start treatment with pazopanib starting on Day 1 at 800 mg po once daily continuous dosing.

[10] **Radioiodine**: Treatment with Radioiodine RAI I-131 therapy starts as per Dose Levels indicated after preparation and scanning Week 6. Pazopanib therapy continues during RAI treatment.

[11] **Dynamic PET imaging**: Baseline scan will be done prior to starting pazopanib therapy and then after 3 weeks or more of pazopanib therapy (Week 4).

[12] **Physical Examination** should include general assessment of organ systems in addition to specific evaluation of cancer related symptoms.

[13] **Adverse Events**: See §§5.2, 5.5, and for Adverse Event details.

[14] **Concomitant Medications**: Concomitant medications will be reviewed for prohibited medications at screening.

[15] **Tumor Imaging**: PET/CT, CT or MRI scan to be performed of applicable sites to assess disease status at Screening, at the end of pazopanib therapy and then every 3 to 6 months or per institutional standards. The same imaging modality(ies) must be used for each patient throughout the study. Imaging should be done when disease progression is suspected; after 4 weeks or more following initial imaging demonstrating either PR or CR to confirm tumor response, and at the time of withdrawal from the study.

[16] **Brain imaging / Bone scan**: Brain CT or MRI should be performed at Screening if brain metastases are suspected. A bone scan should be performed at Screening if bone metastases are suspected.

10.2 APPENDIX B: REQUIRED LABORATORY TESTS

	Conventional Units	Conversion Factor	SI Units
**Hematology**			
Hemoglobin (Hgb)	g/dL	x 10	g/L
Platelet count (Plt)	10^3/mm^3	x 10^9	10^{12}/L
White blood count (WBC)	10^3/mm^3	x 10^6	10^9/L
White blood cell differential	%	x 0.01	fraction
**Chemistry**			
Total bilirubin	mg/dL	x 17.1	μmol/L
Alanine transaminase (ALT)	U/L	N/A	U/L
Aspartate transaminase (AST)	U/L	N/A	U/L
Alkaline phosphatase	U/L	N/A	U/L
Total protein	g/dL	x 10	g/L
Albumin	g/dL	x 10	g/L
Sodium	MEq/L	x 1.0	mmol/L
Potassium	MEq/L	x 1.0	mmol/L
Chloride	MEq/L	x 1.0	mmol/L
Calcium	mg/dL	x 0.25	mmol/L
Blood urea nitrogen (BUN)	mg/dL	x 0.357	mmol/L
Creatinine	mg/dL	x 88.4	μmol/L
Glucose	mg/dL	x 0.055	mmol/L
**Additional Specific tests**			
Thyroglobulin	microIU/mL		mIU/L
TSH	pg/mL		pmol/L
Free T4	ng/mL		pmol/L
Free T3	ng/mL		ug/L

10.3 CLINICAL LABORATORY ASSESSMENTS

Laboratory assessments should be performed as indicated in the Schedule of Protocol Assessments and Events (Appendix A) Table. These assessments may be carried out within 3 days before the actual visit to allow flexibility in scheduling. Assessments may be performed more frequently if clinically indicated. Correction of electrolytes (most importantly, potassium, magnesium and calcium) to within normal ranges should take place prior to study entry and during study conduct as clinically indicated.

All laboratory tests with values that become abnormal and clinically significant while the subject is participating in the study or within 28 days after the last dose of study drug should be repeated until the values return to normal or baseline.

The table below shows the clinical laboratory assessments that should be performed.

Clinical Laboratory Assessments

**Clinical Chemistry**	
Renal function	Urea, Creatinine[a]
Liver function test (LFT) Panel	Albumin, Alkaline phosphatase, Alanine aminotransferase (ALT), Aspartate aminotransferase (AST) and Bilirubin (total)[b]
Electrolytes and others	Calcium, Potassium, Sodium, Glucose, and Lactate Dehydrogenase (LDH)
**Hematology**	Hematocrit, Hemoglobin, White Blood Cell Count, Red Blood Cell Count, Neutrophils, and Platelets
**Coagulation Tests**	Activated partial thromboplastin (aPTT) and International Normalization Ratio (INR)[c]
**Urinalysis for Proteinuria**	UPC[d]
**Thyroid Function Test**	TSH[e]

a) Estimated creatinine clearance should be calculated using the Cockroft and Gault method (Appendix G). Alternatively, creatinine clearance can be measured directly by 24-hour urine collection.
b) A direct bilirubin level should be obtained if the total bilirubin level is greater than 1.5 X upper limit of normal (ULN). See Section 5.14 and 5.14.1 for stopping criteria and dose modification guidelines for treatment-emergent liver function abnormality.
c) Coagulation tests may also be performed in response to an AE/SAE of bleeding and as clinically indicated.
d) UPC should be evaluated as described in Appendix F or by 24-hour urine protein. If UPC ≥ 3 or if urine protein is ≥3g, then the dose modification table guidelines should be followed (Section 5.13.1).
e) Unscheduled thyroid function tests [TSH and thyroxine (free T_4)] should be performed if clinically indicated (e.g., if a subject develops signs and symptoms suggestive of hypothyroidism).

10.4 Appendix C: ECOG Performance Status

Grade	Description
0	Fully active, able to carry on all pre-disease performance without restriction
1	Restricted in physically strenuous activity but ambulatory and able to carry out work of a light or sedentary nature, e.g., light housework or office work
2	Ambulatory and capable of all self-care but unable to carry out any work activities. Up and about >50% of waking hours
3	Capable of only limited self-care, confined to a bed or chair >50% of waking hours
4	Completely disabled. Cannot carry on any self-care. Totally confined to bed or chair
5	Dead

10.5 Appendix D: RECIST Tumor Assessment Criteria (Version 1.1)

The determination of antitumor efficacy during this study will be based on objective tumor assessments made according to the RECIST system of unidimensional evaluation. A minor modification will be adopted to accommodate standard practice in use of spiral CT scan (i.e., reconstruction interval up to 8 mm). In the event spiral CT scan is used to assess tumors, minimum lesion size qualifying as measurable will be twice the reconstruction interval used and at least 10 mm.

Measurability of Tumor Lesions At baseline, individual tumor lesions will be categorized by the Investigator as either measurable or non-measurable by the RECIST criteria as described below.

- Measurable: Lesions that can be accurately measured in at least 1 dimension (longest diameter to be recorded) as ≥ 20 mm with conventional techniques or as ≥ 10 mm with spiral CT scan (depending on reconstruction interval). Clinical lesions will only be considered measurable when they are superficial (e.g., skin nodules, palpable lymph nodes).
- Non-Measurable: All other lesions, including small lesions and bone lesions, leptomeningeal disease, ascites, pleural or pericardial effusions, lymphangitis of the skin or lung, abdominal masses that are not confirmed and followed by imaging techniques, cystic lesions, previously irradiated lesions, and disease documented by indirect evidence only (e.g., by laboratory tests such as alkaline phosphatase).

NOTE: If measurable disease is restricted to a solitary lesion, its neoplastic nature should be confirmed by cytology/histology.

Recording Tumor Measurements

All measurable lesions up to a maximum of 5 lesions per organ and 10 lesions in total representative of all involved organs should be identified as target lesions and measured and recorded at baseline and at the stipulated intervals during treatment. Target lesions should be selected on the basis of their size (lesion with the longest diameters) and their suitability for accurate repetitive measurements (either by imaging techniques or clinically).

The longest diameter will be recorded for each target lesion. The sum of the longest diameter for all target lesions will be calculated and recorded as the baseline sum longest diameter to be used as reference to further characterize the objective tumor response of the measurable dimension of the disease during treatment. All measurements should be performed using a caliper or ruler.

All other lesions (or sites of disease) should be identified as non-target lesions and should also be recorded at baseline. Measurements are not required and these lesions should be followed as “present” or “absent.”

Techniques for Assessing Measurable Disease

The same method of assessment and the same technique should be used to characterize each identified and reported lesion at screening and during follow-up. Imaging-based evaluation is preferred to evaluation by clinical (physical) examination when both methods have been used to assess the antitumor effect of a treatment.

Definitions of Tumor Response

Target Lesions

- Complete response (CR) is defined as the disappearance of all target lesions.

- Partial response (PR) is defined as a 30% decrease in the sum of the longest dimensions of the target lesions taking as a reference the baseline sum longest dimensions.
- Progressive disease (PD) is defined as a 20% increase in the sum of the longest dimensions of the target lesions taking as a reference the smallest sum of the longest dimensions recorded since the treatment started, or the appearance of one or more new lesions.
- Stable disease (SD) is defined as neither sufficient shrinkage to qualify for PR nor sufficient increase to qualify for PD taking as a reference the smallest sum of the longest dimensions since the treatment started.

Non-Target Lesions

- Complete response (CR) is defined as the disappearance of all non-target lesions.
- Incomplete response (SD) is defined as a persistence of 1 non-target lesion.
- Progressive disease (PD) is defined as unequivocal progression of existing non-target lesions, or the appearance of new lesions.

The cytological confirmation of the neoplastic origin of any effusion that appears or worsens during treatment when the measurable tumor has met criteria for response or stable disease is mandatory to differentiate between response or stable disease and progressive disease.

Confirmation of Tumor Response

To be assigned a status of PR or CR, changes in tumor measurements in patients with responding tumors must be confirmed by repeat studies that should be performed ^{3}4 weeks after the criteria for response are first met.

Determination of Overall Response by the RECIST Criteria

When both target and non-target lesions are present, individual assessments will be recorded separately. The overall assessment of response will involve all parameters as depicted below in Table 7.

Table 7 Response Evaluation Criteria in Solid Tumors

**Target Lesions[1]**	**Non-Target Lesions[2]**	**New Lesions[3]**	**Overall Response**
CR	CR	No	CR
CR	Incomplete response/SD	No	PR
PR	Non-PD	No	PR
SD	Non-PD	No	SD
PD	Any response	Yes or No	PD
Any response	PD	Yes or No	PD
Any response	Any response	Yes	PD

[1]Measurable lesions only.
[2]May include measurable lesions not followed as target lesions or non-measurable lesions.
[3]Measurable or non-measurable lesions.

The best overall response is the best response recorded from the start of the treatment until disease progression/recurrence (taking as reference for progressive disease the smallest measurements recorded since the treatment started). The patient's best response assignment will depend on the achievement of both measurement and confirmation criteria.

NOTE: Patients with a global deterioration of health status requiring discontinuation of treatment without objective evidence of disease progression at that time should be reported as "symptomatic deterioration." Every effort should be made to document the objective progression even after discontinuation of treatment.

In some circumstances, it may be difficult to distinguish residual disease from normal tissue. When the evaluation of complete response depends upon this determination, it is recommended that the residual lesion be investigated by fine needle aspirate or biopsy before confirming the complete response status.

10.6 Appendix E: NCI CTC AE

National Cancer Institute (NCI) Common Terminology Criteria for Adverse Events (CTCAE)

The NCI CTCAE (Version 4.03 dated 14 June 2010) has been placed in the Study Reference Binder for this protocol. Alternatively, the NCI CTCAE may be reviewed on-line at the following NCI website: http://ctep.cancer.gov/reporting/ctc.html

10.7 APPENDIX F: URINE PROTEIN CREATININE RATIO (UPC)

Clinical meaning of UPC

There is a good correlation between the ratio of protein concentration to creatinine concentration in a random urine sample and the amount of protein excreted over 24 hours. Creatinine excretion is fairly constant throughout the day regardless of changes in urine flow rate.

Normal protein excretion is <150 mg/24 hours and is similar for men and women.

Men excrete 20 mg to 25 mg of creatinine/kg of body weight/day.

Women excrete 15 mg to 20 mg of creatinine/kg of body weight/day.

Calculating UPC

UPC ratio = Urine protein (mg/dL) / Urine creatinine (mg/dL).
UPC ratio ≈ equivalent to grams of protein excreted in urine over 24 hrs.

Example: Patient has a urine protein = 90 mg/dL and urine creatinine = 30 mg/dL.

UPC ratio= (90 mg/dL) / (30 mg/dL) = 3

The calculated UPC ratio is 3, which correlates to roughly 3 g protein excretion in a 24-hour period.

Units for UPC ratio

Note: To calculate UPC, protein and creatinine concentrations must be expressed in the same units (mg/dL, g/L, or µmol/L). If, for example, protein concentration is expressed in mg/dL and creatinine concentration is expressed in µmol/L, conversion of one of the concentration values is required. Conversion factors are:

From	To	Conversion Factor
Conventional Units: mg/dL	SI Units: µmol/L	Multiply by 88.4
SI Units: µmol/L	Conventional Units: mg/dL	Divide 88.4

References:

Xin G, Wang M, Jian L, Xu F, Wang H. Protein-to-creatinine ratio in spot urine samples as a predictor of quantitation of proteinuria 2004. Clinica Chimica Acta 350:35-39.

NKF: NKF KDOQI Guidelines [Internet]. National Kidney Foundation; nd. KDOQI Clinical Practice Guidelines for Chronic Kidney Disease: Evaluation, Classification, and Stratification. Available from http://www.kidney.org/professionals/KDOQI/guidelines_ckd/p5_lab_g5.htm

10.8 APPENDIX G: DETERMINATION OF CREATININE CLEARANCE (CL_{CR})

Estimation of creatinine clearance using Cockroft and Gault method:

Cl_{CR} for males (mL/min) = $\dfrac{[140 - \text{age (years)}] \times [\text{weight (kg)}]}{(72) \times [\text{Serum creatinine (mg/dL)}]}$

Cl_{CR} for females (mL/min) = $\dfrac{(0.85) \times [140 - \text{age (years)}] \times [\text{weight (kg)}]}{(72) \times [\text{Serum creatinine (mg/dL)}]}$

For SI units:

Cl_{CR} for males (mL/min) = $\dfrac{[140 - \text{age (years)}] \times [\text{weight(kg)}] \times (1.23)}{[\text{Serum creatinine } (\mu\text{mol/L})]}$

Cl_{CR} for females (mL/min) = $\dfrac{[140 - \text{age(years)}] \times [\text{weight(kg)}] \times (1.05)}{[\text{Serum creatinine } (\mu\text{mol/L})]}$

Calculation of creatinine clearance based on 24-hour urinary creatinine excretion and concurrent serum creatinine levels:

$$Cl_{CR} = \frac{C_U \cdot V}{C_{CR}}$$

Here, C_U is the concentration of creatinine in the urine (mg/dL or µmol/L, for SI units), V is the urine volume (in mL per minute of urine produced during the collection period), C_{CR} is the serum creatinine concentration (mg/dL or µmol/L, for SI units), and Cl_{CR} is the creatinine clearance in mL per minute.

10.9 APPENDIX H: SUPPORTIVE CARE GUIDELINES FOR DIARRHEA, NAUSEA, AND VOMITING

These general guidelines are provided to facilitate subject care in the event of diarrhea, thereby avoiding serious complications. Guidelines such as these should never replace sound clinical judgment. Experience thus far suggests that use of monotherapy pazopanib is associated with an increased incidence of diarrhea, primarily of Grade 1 or 2. In rare cases, diarrhea can be debilitating and potentially life threatening, with dehydration, renal insufficiency, and electrolyte imbalances.

Standardized and universal guidelines have been developed by an American Society of Clinical Oncology panel for treating chemotherapy-induced diarrhea [Benson, 2004].

Early identification and intervention is critical for the optimal management of diarrhea. A subject's baseline bowel patterns should be established so that changes in patterns while on treatment can be identified. An assessment of frequency, consistency, and duration of diarrhea, as well as knowledge of other symptoms such as fever, cramping, abdominal pain, nausea, vomiting, dizziness and thirst should be taken at baseline, permitting identification of patients at high risk of diarrhea. Patients should be educated on signs and symptoms of diarrhea with instructions to report any changes in bowel patterns to the study site physician.

The NCI CTCAE Version 4.0 criteria for defining diarrhea are provided below.

Toxicity Grade	Diarrhea (includes diarrhea of small bowel or colonic origin and/or ostomy diarrhea)
1	Increase of <4 stools/day over baseline; mild increase in ostomy output compared to baseline
2	Increase of 4-6 stools/day over baseline; moderate increase in ostomy output compared to baseline
3	Increase of ≥7 stools/day over baseline; incontinence; hospitalization indicated; severe increase in ostomy output compared to baseline; limiting self care activities of daily living
4	Life threatening consequences, urgent intervention indicated
5	Death

Uncomplicated diarrhea is considered mild to moderate and is defined as CTCAE Grade 1 to 2 with no complicating signs or symptoms.

Complicated diarrhea is severe and defined as CTCAE Grade 3 or 4 or Grade 1 or 2 with one or more of the following signs or symptoms: severe cramping, ≥Grade 2 nausea/vomiting, decreased performance status, fever, sepsis, Grade 3 or 4 neutropenia, obvious bleeding, dehydration.

Management Guidelines

Uncomplicated diarrhea of CTCAE Grade 1 or 2:

- Hydration: have subject drink 8 to 10 large glasses (approximately 2 liters) of clear non-caffeinated liquids a day (e.g., broth or electrolyte-containing sports drinks).
- If Grade 2 diarrhea, consider dose reduction of investigational products.
- Dietary modifications: have subject stop all lactose-containing products and eat frequent, small meals
- Pharmacologic intervention using loperamide:
    - Begin loperamide at initial dose of 4 mg followed by 2 mg every 4 hours or after every unformed stool. The recommended maximum daily dose of loperamide is 16 mg/day.

- Continuation of loperamide is suggested until diarrhea-free for 12 hours.
- If mild to moderate diarrhea persists for more than 24 hours, administer loperamide 2 mg every 2 hours and pursue evaluation for other treatable causes.
- If mild to moderate diarrhea persists after 48 hours total treatment with loperamide, discontinue study drug(s) and consider initiation of second-line agents (lomotil, octreotide).

Complicated diarrhea of CTCAE Grade 3 or 4 diarrhea or Grade 1 or 2 with complicating features requires aggressive management:

- Subject must call study site physician immediately in response to any event of severe diarrhea with or without complications as listed above.
  - Hospitalization may be required for subjects most at risk for life-threatening complications.
- Interrupt investigational products until symptoms resolve; consider reintroducing at a reduced dose (discuss with GSK Medical Monitor or designee).
- If loperamide has not been initiated, begin loperamide usage immediately at an initial dose of 4 mg followed by 2 mg every 2 hours or after every unformed stool. The recommended maximum daily dose of loperamide is 16 mg/day.
- If no improvement in severity after 24-hours of maximal loperamide dosing, subject must visit study site and be evaluated:
  - For dehydration, use intravenous fluids as appropriate.
- Antibiotic therapy should be considered in patients, who present with signs and symptoms of bacterial diarrhea such as fever, bloody diarrhea, and presence of fecal leukocytes. Investigators should have a low threshold to start such treatment in patients with Grade 3 or Grade 4 neutropenia.
- Before initiation of antimicrobial therapy, stool cultures should be obtained. When bacterial etiology for diarrhea is suspected, study-treatment and anti-motility agents (loperamide or others) should be held.
- Intervention should be continued until diarrhea free for 24 hours.

Alternative Pharmacologic Intervention for Uncomplicated and Complicated Diarrhea

- Lomotil (dephenoxylate 2.5 mg + atropine 0.025 mg) can be used. The recommended dose is 2 tablets 4 times daily. When diarrhea is under control, a dose reduction should be attempted.
- The synthetic octapeptide, octreotide, has been shown to be effective in the control of diarrhea induced by flouropyrimidine-based chemotherapy regimens when administered as an escalating dose by continuous infusion or subcutaneous injection. Octreotide can be administered at doses ranging from 100 μg twice daily to 500 μg 3 times daily, with a maximum-tolerated dose of 2000 μg 3 times daily in a 5-day regimen.

Nausea and Vomiting

Every attempt should be made to control nausea and vomiting in subjects who have emesis and are unable to retain pazopanib.

Routine pre-medication for nausea is not necessary, but symptomatic subjects should be treated with standard anti-nausea/anti-emetic therapy as necessary.

If a subject vomits after taking study medication, the subject should be instructed not to take a replacement dose on that same day. The subject should resume taking pazopanib at the next

scheduled dose on the following day. If vomiting persists, then the subject should contact their physician.

To prevent or treat nausea and vomiting standard medications are recommended. Depending upon approved medications in your region, these may include: 5-HT_3 receptor antagonist (granisetron, ondansetron, dolasetron mesylate); NK-1 receptor antagonists such as aprepitant, metoclopramide, phenothiazines (prochlorperazine); corticosteroids, (dexamethasones, prednisone); and cannibinoids (dronabinol).

Reference:

Benson AB, Ajani JA, Catalano RB, Engelking C, Kornblau SM, Martenson JA, et al., Recommended Guidelines for the Treatment of Cancer-Induced Diarrhea. J Clin Oncol. 2004, 22; 2918-26.
